# Perfluorocarbon regulates the intratumoural environment to enhance hypoxia-based agent efficacy

**DOI:** 10.1038/s41467-019-09389-2

**Published:** 2019-04-05

**Authors:** Wenguang Wang, Yuhao Cheng, Peng Yu, Haoran Wang, Yue Zhang, Haiheng Xu, Qingsong Ye, Ahu Yuan, Yiqiao Hu, Jinhui Wu

**Affiliations:** 1 State Key Laboratory of Pharmaceutical Biotechnology, Medical School of Nanjing University and School of Life Sciences, Nanjing University, 210093 Nanjing, China; 2 Institute of Drug R&D, Nanjing University, 210093 Nanjing, China; 3 Jiangsu Provincial Key Laboratory for Nano Technology, Nanjing University, 210093 Nanjing, China

## Abstract

Hypoxia-based agents (HBAs), such as anaerobic bacteria and bioreductive prodrugs, require both a permeable and hypoxic intratumoural environment to be fully effective. To solve this problem, herein, we report that perfluorocarbon nanoparticles (PNPs) can be used to create a long-lasting, penetrable and hypoxic tumour microenvironment for ensuring both the delivery and activation of subsequently administered HBAs. In addition to the increased permeability and enhanced hypoxia caused by the PNPs, the PNPs can be retained to further achieve the long-term inhibition of intratumoural O_2_ reperfusion while enhancing HBA accumulation for over 24 h. Therefore, perfluorocarbon materials may have great potential for reigniting clinical research on hypoxia-based drugs.

## Abstract (web summary)

Hypoxia-based agents need permeable and hypoxic intratumour environment to be effective. Here, the authors show that perfluorocarbon nanoparticles promote increased permeability and sustained hypoxia to improve accumulation of hypoxia-based agents, and inhibit intratumour oxygen reperfusion.

## Introduction

Hypoxia-based agents (HBAs) exert hypoxia-based cytotoxic effects against solid tumours^1,2^. For example, the bioreductive prodrug tirapazamine (TPZ) can be reductively metabolized to toxic reductants in hypoxic tumours; Salmonella and other facultative anaerobic bacteria can selectively damage hypoxic cancer cells via pathogenicity island 2 and the immune response of the host^3–11^. However, these anaerobic therapies ultimately failed due to unsatisfactory efficacy in clinical studies. TPZ, for instance, clinically failed due to restricted penetration into hypoxic tumours^12,13^. Since the abnormal vasculature and dense tissue of tumours severely limit intratumoural penetration, those anaerobic drugs are generally restricted to the normoxic tumour periphery rather than allowed to reach the hypoxic core^14–16^. As a result, HBAs have little effect on hypoxic or aerobic cells in whole tumours.

Both the periphery and hypoxic core of tumours are less permeable^16^; therefore, the penetration of HBAs can be optimized by improving tumour permeability for treating whole tumours. However, increased tumour permeability achieved using antiangiogenic agents^17^ and other methods are also associated with the reperfusion of O_2_^18–22^, which further weakens the effect of HBAs. Conversely, other methods that aim to increase the hypoxia of tumours, such as blocking tumour capillaries, are associated with low drug penetration^23^. As such, photodynamic therapy (PDT), which both consumes intratumoural oxygen and enhances tumour permeability, has recently been utilized to enhance the efficacy of HBAs; however, the efficacy could not be maintained after the laser was removed^24^. Therefore, there is still an urgent need for new approaches to enhance the efficacy of HBAs.

**Fig. 1:**
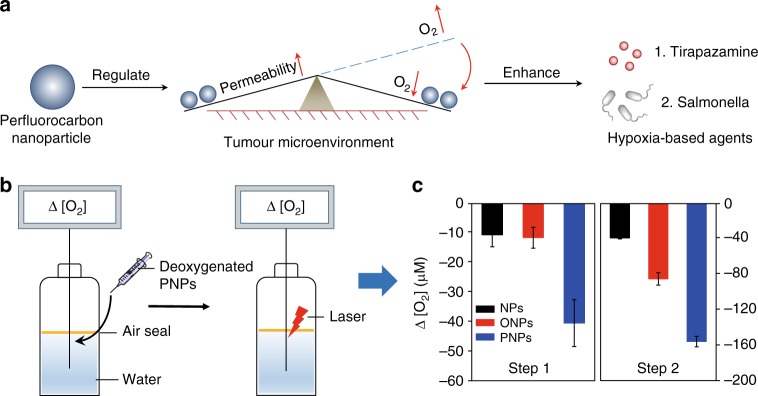
A schematic diagram of PNP-mediated improvements in the function of HBAs and O_2_ absorption and consumption by PNPs. **a** Schematic diagram of PNP-enhanced hypoxia-based drug efficacy. PNPs enable both enhanced tumour permeability and long-term hypoxia to improve the efficacy of HBAs. **b** Schematic illustration of the measurement of the O_2_ absorption/consumption ability of the PNPs. Step 1: Deoxygenated samples were added to H_2_O sealed with oil; changes in DO (dissolved O_2,_ Δ [O_2_]) were measured using an inserted O_2_ probe. Step 2: Changes in DO before and after laser irradiation. **c** Quantitative determination of DO variations in steps 1 and 2 (*n* = 3). Data are presented as the mean ± s.e.m. Source data are provided as a Source Data file

To promote drug-rich and oxygen-free conditions in the tumour environment, we report the use of a clinically approved perfluorocarbon, named perfluorotributylamine (PFTBA)^25–29^, to simultaneously enhance tumour permeability and intratumoural hypoxia to match the requirements of HBAs. First, tumour permeability can be enhanced by PFTBA due to its platelet inhibition effects^30–33^. Subsequently, tumour deoxygenation is achieved by PFTBA absorbing oxygen and by PDT. Third, in contrast to previous PDT agents, retained PFTBA can further maintain the favourable environmental conditions for over 24 h to enable maximal HBA delivery and activation via the oxygen-absorbing and platelet-inhibiting activities of PFTBA (Fig. 1). The enhanced efficacy of combinations of PFTBA nanoparticles (PNPs) and two typical HBAs (TPZ and Salmonella VNP20009 (Sa.)) was confirmed in vivo. In summary, this work highlights the promise of anaerobic therapies using perfluorocarbon to establish a long-lasting, hypoxic and permeable tumour microenvironment.

## Results

### Preparation and characterization of PNPs

PNPs were prepared by an unfolding/self-assembling method previously reported by our group^34–36^. Briefly, PFTBA was ultrasonically emulsified with an IR780 (a near-infrared (NIR) photosensitizer)-loaded human serum albumin (HSA) to form PNPs. IR780 nanoparticles (NPs) and oil nanoparticles (ONPs) were also prepared by replacing PFTBA with water or olive oil as controls. The structure of the PNPs was clearly visualized as a uniform sphere-like structure using transmission electron microscopy (Supplementary Fig. 1a). By dynamic light scattering (DLS) (Supplementary Fig. 1b), the average PNP diameter was determined to be 160 nm, and this value did not change after 7 days of storage (Supplementary Fig. 2a), indicating the long-term stability of the PNPs in phosphate-buffered saline (PBS). The quantity of IR780 in the PNPs was also determined by the ultraviolet–visible (UV–vis) absorption spectra according to the standard curve of IR780 (Supplementary Fig. 2b).

Perfluorocarbons, such as PFTBA, all have stronger oxygen-absorbing abilities than water (Supplementary Figs. 3, 4), which can enhance the photodynamic efficacy of surrounding photosensitizers, as we have previously reported^37^. Considering that tumours can continually receive oxygen from the circulation, sustained oxygen consumption is quite important for inhibiting the recovery of the partial pressure of oxygen in tumours. PFTBA can absorb higher levels of dissolved oxygen (DO) and thus may meet this goal. To verify whether PNP can serve as such an oxygen nanosponge, we added deoxygenated PNPs to water to simulate the process of O_2_ absorption (step 1, Fig. 1b). We found that a remarkable Δ [O_2_] in the PNP solution (−41.7 μM), but only slight Δ [O_2_] in the ONP and NP solutions (<−12 μM, step 1, Fig. 1c). After O_2_ absorption in step 1, the PNPs were irradiated with an 808 nm laser to achieve oxygen absorption and consumption (step 2, Fig. 1b). The Δ [O_2_] value was further decreased in the PNP group, and the decline was much greater in the PNP group than in the control groups (−160 μM for PNPs, −90μM for ONPs and −40 μM for NPs) (step 2, Fig. 1c). These results indicated that the PNPs absorbed and consumed oxygen. To further assess the laser-stimulated deoxygenated PNPs, we measured the Δ [O_2_] value of a solution of irradiated PNPs without any pre-deoxygenation (Supplementary Fig. 5a). The Δ [O_2_] value decreased much more in the PNP group than in the control groups (Supplementary Fig. 5b). This decline in DO was also confirmed using an O_2_-sensitive phosphorescent molecular probe (Mito-Xpress Kit)^38,39^ (Supplementary Fig. 6).

We further evaluated whether the above light/dark cycle could enhance hypoxia repeatedly. The PNP group presented a more effective reduction in O_2_ than did the ONP group. After four cycles, the PNPs could still uptake and consume O_2_ effectively (Supplementary Fig. 7). These observations suggest that PNPs have a sustainable and repeatable oxygen absorption and consumption capacity.

### Long-term enhancement of permeability and hypoxia via PNPs in vivo

In addition to the in vitro regulation of tumour hypoxia, we studied the behaviours of deoxygenated PNPs in regulating tumour hypoxia and permeability in vivo. Considering that the accumulation and action of hypoxic drugs often take hours or even days (such as for anaerobic bacteria), it is critical for such deoxygenated PNPs to enhance permeability and hypoxia for a long time.

**Fig. 2:**
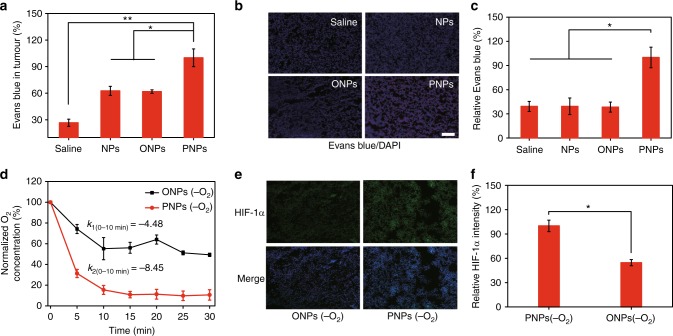
Long-term effects of enhanced permeability and hypoxia mediated by deoxygenated PNPs in vivo. **a** Analysis of Evans blue accumulation in tumours 24 h after the intravenous injection of deoxygenated PNPs. Evans blue in the whole tumour was extracted and quantified by ultraviolet–visible (UV–vis) spectroscopy (*n* = 3). **b** Ex vivo fluorescence images of Evans blue (red) and 4′,6-diamidino-2-phenylindole (DAPI) (blue) staining (scale bar, 100 µm). **c** Quantification of Evans blue accumulation in **b** (*n* = 3). **d** Changes in DO after the intratumoural injection of deoxygenated PNPs and oil nanoparticles (ONPs). **e** Representative immunofluorescence images of hypoxia-inducible factor-1α (HIF-1α) (green) staining at 24 h after the intratumoural injection of deoxygenated PNPs and ONPs (scale bar, 100 µm). **f** Quantification of HIF-1α expression shown in **e** (*n* = 4). Data are presented as the mean ± s.e.m. *p *<* 0.05, **p < 0.01 (unpaired, two-way *t* tests). Source data are provided as a Source Data file

As such, we then studied the PNP-mediated regulation of tumour permeability. It is worth noting that tumours and the surrounding vasculature are rich with platelets, which can cause poor drug penetration by promoting tumour tissue densification^40–44^. We have previously reported that inhibiting platelet aggregation can increase tumour permeability^33^. Considering that one of the side effects of perfluorocarbon-based blood substitutes is the inhibition of platelet activity^45^, we speculated whether tumour permeability could be increased by PFTBA, which has a strong inhibitory effect on platelets (Supplementary Fig. 8). The in vivo accumulation of Evans blue, a fluorescent probe that can form self-assembled NPs with HSA and emit red fluorescence, was then tested to evaluate tumour permeability. Evans blue was extracted from the whole tumour using formamide and quantified by the UV–vis absorbance at 620 nm. Significantly more Evans blue was detected in PNP-treated tumours than in tumours treated with saline, NPs, or ONPs (Fig. 2a). Fluorescence imaging and the corresponding quantitative analysis further confirmed that intravenously administered PNPs alone could increase drug permeation (Fig. 2b, c). The tumour penetration of HBA (TPZ) was also significantly increased after PNP treatment compared with the control treatments (Supplementary Fig. 9).

In addition, we examined whether O_2_ reperfusion could be inhibited by the O_2_-absorbing capacity of deoxygenated PNPs (by nitrogen) in vivo. Real-time changes in the intratumoural DO level were recorded after mice were locally injected with deoxygenated PNPs (Fig. 2d). The DO level in the PNP-treated tumours decreased by 80%, while that in the controls decreased by only 40%, indicating that deoxygenated PNPs can inhibit the recovery of intratumoural DO. To assess the O_2_ absorption of deoxygenated PNPs, we also calculated the rate of O_2_ absorption; this rate was higher for the PNPs (*k*_2_, −8.45) than the controls (*k*_1_, −4.48), further indicating that deoxygenated PNPs could more efficiently absorb the surrounding O_2_ to create a more hypoxic microenvironment.

Hypoxia-inducible factor-1α (HIF-1α) staining was performed 24 h after injection. Significant green fluorescence was observed in tumours treated with deoxygenated PNPs, whereas cells treated with ONPs still showed negligible fluorescence (Fig. 2e). Quantitative analysis of the HIF-1α expression in tumour tissue treated with deoxygenated PNPs showed double the expression observed in tumour tissue treated with ONPs (Fig. 2f), suggesting that PNPs could actively extract surrounding O_2_, resulting in long-lasting tumour hypoxia.

### Irradiation of deoxygenated PNPs to regulate permeability and hypoxia

To evaluate the effect of laser irradiation on the observed hypoxia, the effects of deoxygenated PNPs on hypoxia and permeability after laser stimulation were evaluated in vitro. Enhanced cellular hypoxia was first confirmed using a hypoxia stress detection kit. The results showed that the hypoxia signals in cells treated with laser-stimulated PNPs were significantly increased compared to those in cells treated with NPs and ONPs (Supplementary Fig. 10a). Cellular hypoxia was also confirmed using anti-HIF-1α antibody (Supplementary Fig. 10b), suggesting that longer-lasting hypoxic environments could be created by the stimulated PNPs with a laser. The in vitro generation of singlet oxygen (^1^O_2_) under different O_2_ environments was confirmed, and the cellular ^1^O_2_ level was determined (Supplementary Fig. 10c, 11). The laser stimulation of PNPs also resulted in the best cytotoxicity among all treatments (Supplementary Fig. 12); this approach might enhance tumour penetration by severely damaging the dense tumour tissue.

To explore the distribution of the PNPs in vivo, intravenously dosed mice were examined by NIR imaging at 12, 24 and 36 h post administration (Supplementary Fig. 13). PNPs emitted the strongest fluorescence in tumours 24 h post injection, and this time point was chosen for performing laser irradiation if needed. Laser-stimulated ^1^O_2_ generation in vivo was then detected using 2′, 7′-dichlorodihydrofluorescein diacetate (H_2_DCFDA) (Supplementary Fig. 14). In addition, the effect of photothermal treatment was evaluated in vivo. No obvious temperature increase sufficient to effectively ablate tumours was observed in the PNP or NP groups (Supplementary Fig. 15).

**Fig. 3:**
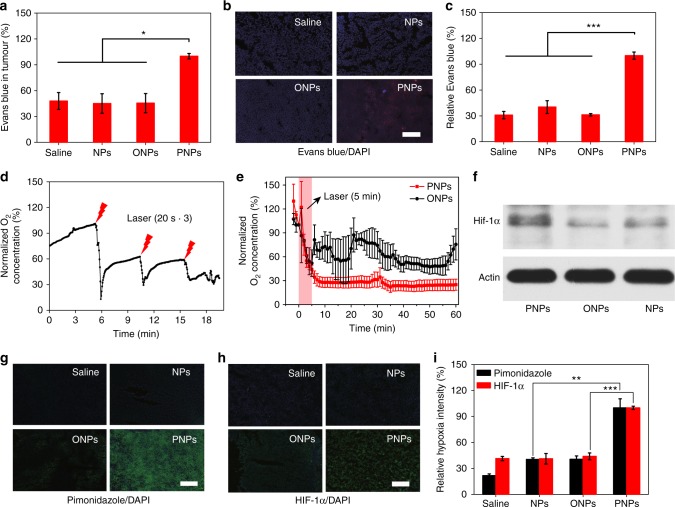
Increased tumour permeability and hypoxia mediated by deoxygenated PNPs under laser irradiation. **a** The accumulation of Evans blue in tumours was quantified 24 h after the intravenous injection of PNPs and 3 h after laser irradiation (5 min, 808 nm, 400 mW cm^−2^), (*n* = 3). **b** Ex vivo fluorescence images of Evans blue (red) in tumour sections from **a**. Nuclei were stained with 4′,6-diamidino-2-phenylindole (DAPI) (blue), (scale bar, 100 µm), (*n* = 3). **c** Relative quantification of Evans blue accumulation in **b** (*n* = 4). **d** Changes in dissolved oxygen (DO) during three cycles of laser irradiation (808 nm, 2 W cm^−2^, 20 s) after intratumoural administration. **e** Changes in DO after laser irradiation (808 nm, 400 mW cm^−2^) for 5 min at 24 h post-intravenous injection (*n* = 3). **f** Western blot of intratumoural hypoxia-inducible factor-1α (HIF-1α) expression at 24 h after laser irradiation. **g**, **h** Tumour sections were stained with anti-pimonidazole antibody (green), anti-HIF-1α antibody (green) and DAPI (blue) at 24 h post irradiation (scale bars, 200 µm). **i** Quantification of pimonidazole and HIF-1α staining (*n* ≥ 3). Data are presented as the mean ± s.e.m. *p *<* 0.05, **p < 0.01 and ***p < 0.001 (unpaired, two-way *t* tests). Source data are provided as a Source Data file

To evaluate the effects of irradiated PNPs on HBA permeation in vivo, Evans blue was employed as a fluorescent probe to reflect tumour permeability. In addition to the platelet inhibition of PNPs, in vivo ^1^O_2_ production was expected to cause tumour tissue damage, thus further improving drug accumulation and permeation through vascular infiltration, as well as tumour apoptosis and necrosis. Evans blue was extracted from whole tumour and analysed 3 h post irradiation (Fig. 3a). Twice as much Evans blue was present in tumours treated with PNPs than in tumours treated with saline, NPs or ONPs. Tumour sections (Fig. 3b) and the corresponding quantified data (Fig. 3c) also suggested that PNP-mediated PDT significantly increased the penetration of Evans blue. Therefore, tumour permeability could be further improved with the application of PDT.

To evaluate the effects of laser-irradiated PNPs on the tumour hypoxia status, in vivo real-time oxygen monitoring was employed to reflect the change in DO post irradiation. Tumour hypoxia caused by O_2_/^1^O_2_ conversion was explored in situ using a real-time O_2_ microelectrode inserted into the tumour. We first evaluated whether PNP-based oxygen consumption occurred in a laser-responsive manner. Exposure to a 2 W cm^−2^ 808 nm laser for 20 s was used to study the triggered hypoxia, and changes in intracellular O_2_ were monitored immediately after PNPs were injected into mice intratumourally. DO in the tumours was significantly decreased after laser exposure. Additionally, the DO level would recover immediately after cessation of the short-term laser irradiation, mainly due to the incomplete activation of PNPs (Fig. 3d).

Considering the potential for skin damage with high-intensity laser irradiation, we then applied 400 mW cm^−2^ laser irradiation for 5 min to create a hypoxic environment within the tumour. Similarly, PNPs could significantly decrease the tumour O_2_ level, resulting in more hypoxia than observed in the other groups (Fig. 3e). Interestingly, we found that the DO level continued to decline even after the laser had been removed and did not recover for a long period. This may be due to the transient occlusion of the oxygen supply from the circulation by the damage of tumour vessels (Supplementary Fig. 16) and the oxygen absorption of deoxygenated PFTBA (Fig. 2d–f).

Next, we investigated whether laser-stimulated PNPs could be used to maintain tumour hypoxia for longer periods, so we performed pimonidazole and HIF-1α staining at 24 h post irradiation. Pimonidazole can form protein adducts only under hypoxic conditions, and the hypoxia intensity is then indicated by green fluorescence. Significantly enhanced green fluorescence was observed in the PNP group compared with the control groups (Fig. 3g). Similarly, the HIF-1α fluorescence was significantly stronger in the PNP-treated tumours than in those treated with NPs (Fig. 3h). The quantitative results showed that the hypoxia intensity (pimonidazole and HIF-1α) in PNP-treated tumour sections was two times higher than that in ONP-treated tumour sections (Fig. 3i), indicating the superior ability of PNPs to create and maintain tumour hypoxia after laser irradiation.

Western blotting was also used to investigate tumour hypoxia at 24 h after the treatment. The results showed that, after laser irradiation, the HIF-1α expression in the PNP-treated tumours was much higher than that in the control groups (Fig. 3f). The quantitative results showed that the expression of HIF-1α in the PNP group was almost 2 times as much as that in the ONP group (Supplementary Fig. 17), indicating that PNPs could create and maintain long-term tumour hypoxia for at least 24 h in vivo.

### PNPs enhanced TPZ chemotherapy

**Fig. 4:**
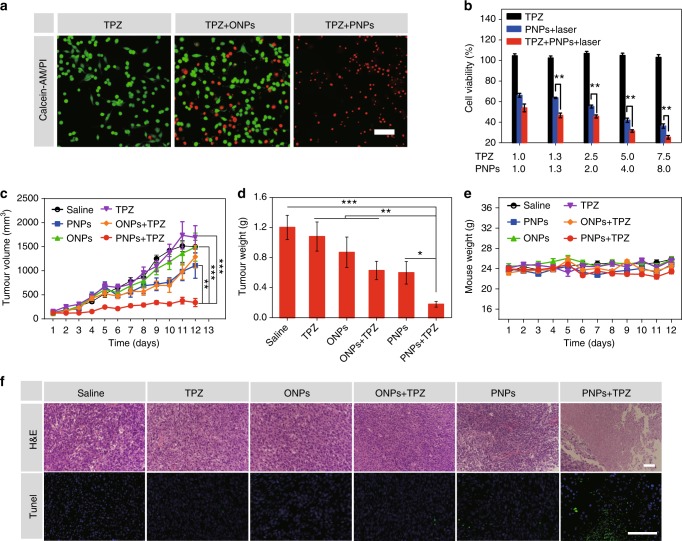
PNPs enhanced TPZ chemotherapy. **a** Fluorescence images of CT26 cells cultured with TPZ and oil nanoparticles (ONPs) or PNPs under laser irradiation (808 nm, 400 mW cm^−2^). Viable cells were stained with Calcein-AM (green), and dead/late apoptotic cells were stained with propidium iodide (PI) (red), (scale bar, 100μm). **b** Viability of treated cells (*n* = 3). **c** Tumour growth curves of treated mice (*n* ≥ 6 per group). Both PNPs and TPZ were injected intravenously into mice on days 1, 3 and 5. Tumours in groups 3, 4, 5 and 6 were irradiated three times (808 nm, 400 mW cm^−2^) for 5 min at 24 h after the administration of the PNPs. **d** Normalized averages of the tumour weight on day 12. **e** Changes in body weight. **f** Haematoxylin and eosin (H&E) (scale bars, 100 µm) and terminal deoxynucleotidyl transferase dUTP nick-end labelling (TUNEL) (green) (scale bars, 100 µm) staining of CT26 tumour sections. Samples were collected from different groups on day 8 post administration. Data are presented as the mean ± s.e.m. *p < 0.05, **p < 0.01, *** *p* < 0.001 (unpaired, two-way *t* tests). Source data are provided as a Source Data file

To explore whether the PNPs could synergistically improve the efficacy of HBAs in treating tumours, we chose TPZ, a widely studied bioreductive prodrug, as a typical HBA. We applied laser-stimulated PNPs to build a permeable and hypoxic environment that could be maintained long enough for the in vivo study and was easy to achieve. Calcein-AM/propidium iodide (PI) apoptosis detection was first performed to measure the apoptosis-inducing capability of the HBA plus the PNPs in vitro. All cells showed green fluorescence before laser irradiation, indicating that both the PNPs and TPZ were non-toxic (Supplementary Fig. 18). After irradiation, cells treated with PNPs plus TPZ displayed stronger red fluorescence, whereas those treated with ONPs plus TPZ showed relatively weak red fluorescence (Fig. 4a), indicating the optimal cytotoxicity of TPZ plus PNPs with laser exposure.

Staining with Alamar Blue was then performed to confirm the cytotoxicity in vitro. Few cells incubated with TPZ were damaged after laser irradiation (Fig. 4b). Cells treated with PNPs plus TPZ showed significant cytotoxicity (46.6 and 25.2%), whereas relatively moderate cytotoxicity (63.6 and 36.2%) was observed in tumours treated with PNPs alone when the concentration of IR780 was 1.3 and 7.5 μg mL^−1^, respectively. These results further indicated that PNP-mediated PDT could effectively increase the cytotoxicity of TPZ due to the hypoxic environment caused by PNPs. TPZ has no cytotoxic effects under normoxic conditions and only shows toxicity under hypoxic conditions (Supplementary Fig. 19). As PNPs continuously absorb and consume the surrounding O_2_, TPZ is converted into toxic radicals and damages tumour cells more effectively.

We then evaluated the enhanced efficacy of the combined therapy by pretreating CT26 tumour-bearing mice with PNPs followed by intratumoural injections of TPZ and monitoring the tumour volume. Tumours treated with PNPs plus TPZ were significantly inhibited compared with tumours in the other groups, which suggested the successful destruction of tumour cells by PNPs combined with TPZ (Supplementary Figs. 20a, b, 21). The results of haematoxylin and eosin (H&E) and terminal deoxynucleotidyl transferase dUTP nick-end labelling (TUNEL) staining also indicated that pretreatment with PNPs significantly enhanced the efficacy of TPZ (Supplementary Fig. 20d). Furthermore, no obvious changes in body weight or tissue damage in normal organs were observed, suggesting that the combined therapy has few side effects (Supplementary Figs. 20c, 22).

The antitumour efficacy of the PNP-enhanced chemotherapy with laser irradiation was then assessed in vivo after the intravenous administration of both PNPs and TPZ. CT26 tumour-bearing mice were randomly divided into six groups: the control (saline), PNP, ONP, TPZ, ONP plus TPZ and PNP plus TPZ group. At 24 h post injection, all tumours were exposed to an 808 nm laser for 5 min at a density of 400 mW cm^−2^. The fastest tumour growth was detected in the saline group, while moderately restricted tumour growth was observed in the PNP and ONP plus TPZ groups. In the TPZ plus PNP group, complete tumour inhibition was achieved (Fig. 4c, Supplementary Fig. 23, 24). The mice were sacrificed on day 13, and the excised tumours were weighed. These results also showed the strongest tumour inhibition in the PNP plus TPZ group (Fig. 4d). The body weight of the mice did not significantly change with any treatment (Fig. 4e), indicating that PNP-based PDT enhanced the anticancer efficacy of TPZ without causing additional toxicity.

H&E and TUNEL staining was also performed to investigate the level of tumour apoptosis ex vivo. H&E staining showed prominent tumour cell necrosis after treatment with PNPs plus TPZ, whereas moderate damage was achieved by PNPs and TPZ alone (Fig. 4f). The TUNEL assay also showed the highest level of apoptosis in tumours treated with PNPs plus TPZ, further confirming the excellent antitumour performance of the combination of TPZ and PNPs.

### PNPs enhanced hypoxia-based bacterial cancer therapy

After successfully demonstrating the effect of TPZ combined with PNPs, Sa. was chosen as another example for combination with PNP pretreatment. A phase I clinical study of VNP20009 failed because of the lack of therapeutic efficacy^7^. Some studies have shown that Sa. only distributes in the necrotic areas of hypoxic tumours, which are far from tumour vessels^5^. In this case, sustained whole-tumour hypoxia is quite important for ensuring that injected bacteria accumulate as much as possible throughout the entire tumour, extensively distributing and proliferating in time and space.

**Fig. 5:**
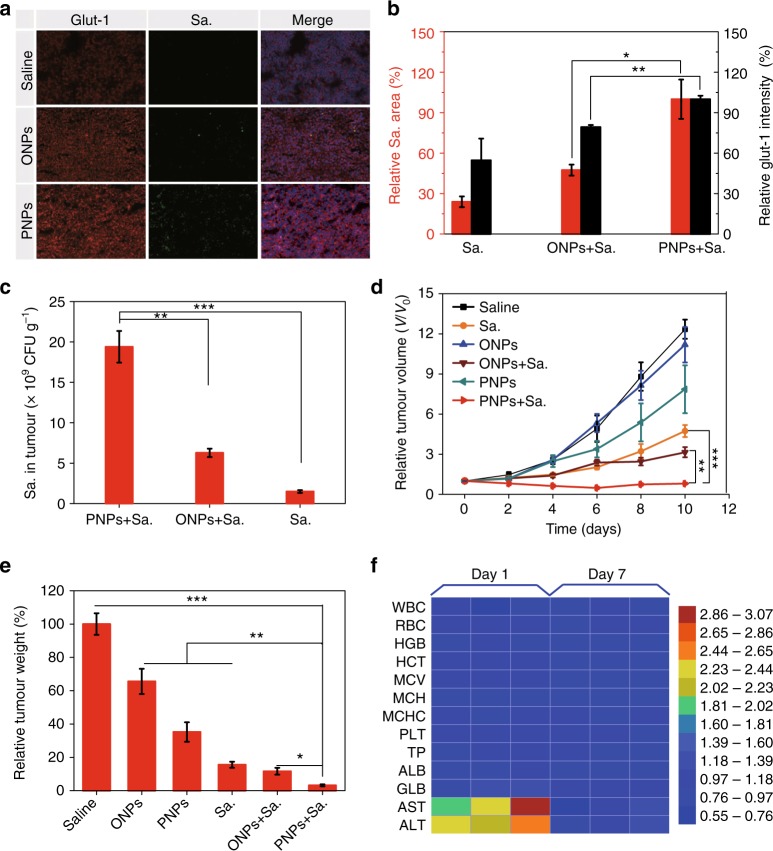
PNPs enabled hypoxia-based bacterial cancer therapy. **a** The hypoxia intensity, Salmonella VNP20009 (Sa.) accumulation and nuclei were determined by staining with anti-glut-1 antibody (red), anti-Sa. antibody (green) and 4′,6-diamidino-2-phenylindole (DAPI) (blue), respectively (scale bar, 100 µm). Sa. (5 × 10^6^ CFU) was intravenously administered to mice 1 h before irradiation. The mice were exposed to the laser (808 nm, 400 mW cm^−2^) for 5 min at 24 h after they were intravenously injected with PNPs, oil nanoparticles (ONPs) or saline. Immunofluorescence images of tumour sections were analysed on day 3 post irradiation (*n* ≥ 3). **b** Semiquantitative analysis of the relative hypoxia intensity (the black *y*-axis) and Sa. distribution (the red *y*-axis) in **a**. **c** The distribution of Sa. in tumours was also measured by colony-forming assay on day 3 (*n* = 3). **d** Tumour growth curves of mice subjected to different treatments (*n* = 4–9). **e** Normalized tumour weight at day 10 post treatment. **f** Haematologic indexes and blood biochemistry of mice that were intravenously injected with VNP20009. The experiment was carried out on days 1 and 7 after the injection of VNP20009 (5 × 10^6^ CFU mouse^−1^, *n* = 3) and saline. Values are the mean ± s.e.m. *p *<* 0.05, **p < 0.01, ***p < 0.001 (unpaired, two-way *t* tests). Source data are provided as a Source Data file

To confirm whether pretreatment with PNPs could enhance the efficacy of the bacteria, we first investigated the intratumoural distribution of VNP20009 by performing ex vivo immunofluorescence staining with glut-1 protein antibody (red) and Salmonella antibody (green). At day 3, sections of PNP-treated tumours showed obviously increased hypoxia levels and VNP20009 distributions compared with the control groups, and VNP20009 was exactly distributed in the hypoxic areas exhibiting greater glut-1 protein expression (Fig. 5a). Semiquantitative analysis of the glut-1 protein and VNP20009 levels revealed that the percentage of the VNP20009 distribution was consistent with the level of glut-1 protein expression (Fig. 5b), demonstrating that PNPs can expand and reinforce the hypoxic zone for to support the infiltration and proliferation of VNP20009.

Next, we evaluated the intratumoural VNP20009 level by colony-forming assay (CFU). Approximately 12.7 times as much VNP20009 accumulated in tumours treated with PNPs plus VNP20009 as in tumours treated with VNP20009 alone (Fig. 5c). In addition, this level was almost 1000 times greater than that in other organs, including the spleen, liver, kidneys, heart and lungs (Supplementary Fig. 25). These results confirmed that PNPs could guide VNP20009 to penetrate and proliferate within tumours more effectively.

Next, we studied the in vivo efficacy of PNP-enhanced bacterial cancer therapy in CT26 tumour-bearing mice. Male BALB/c mice bearing CT26 tumours ~60–80 mm^3^ in size were randomly divided into the following six groups: the saline, PNP, ONP, Sa., ONP plus Sa., and PNP plus Sa. groups. At 24 h post injection, Sa. was intravenously injected if needed; then, all mice were irradiated with an 808 nm laser (400 mW cm^−2^) for 5 min. PNPs plus Sa. achieved the most effective tumour inhibition (Fig. 5d). The mice were sacrificed on day 10, and the tumours were excised, photographed and weighed (Fig. 5e, Supplementary Fig. 26). These experiments also confirmed that PNPs plus Sa. inhibited tumour growth the most. The mice were also weighed (Supplementary Fig. 27). Collectively, these results show that the hypoxic and permeable environment created by PNPs could also improve the effect of hypoxia-based bacterial cancer therapies.

While the safety of such intravenous bacterial treatments has been previously tested^46–49^, we analysed the blood biochemical and haematologic indexes of the mice to assess the safety of our therapy. The blood biochemistry of mice that were injected with VNP20009 intravenously varied significantly on day 1, especially the AST and ALT levels, which were ~2.94 and 2.54 times those in mice treated with saline; however, no long-term side effects of VNP20009 were observed on day 7 post injection (Fig. 5f). These results indicate that the intravenous injection of VNP20009 at a dose of 5 × 10^6^ CFU per mouse is feasible and safe.

## Discussion

HBAs (i.e., TPZ, TH-302, AQ4N and VNP20009) alone usually cannot achieve satisfactory antitumour effects^50–55^. To treat tumours effectively, HBAs face at least three obstacles. First, the dense perivascular tissues are not only insensitive to HBAs but also prevent HBAs from penetrating hypoxic regions. Second, after drug penetration is improved (e.g. via increasing vascular permeability using tumour necrosis factor-α, promoting the normalization of tumour vessels using losartan, or degrading the extracellular matrix (ECM) using matrix metalloproteinases and relaxin), the accompanied reperfusion of blood oxygen will ease the hypoxia and limit drug efficacy. Lastly, the intratumoural distribution of hypoxic drugs often takes hours or even days (such as for Sa.); even if the hypoxic environment and drug penetration are simultaneously increased (i.e. by photodynamic O_2_ to cytotoxic ^1^O_2_ conversion), additional strategies are still needed to maintain these environmental conditions for long periods of time.

In this study, we construct a perfluorocarbon-based nanotherapeutic that can enhance anaerobic therapies in a two-step manner. First, drug permeation and tumour hypoxia were improved via PDT. Synergism between the co-encapsulated photosensitizer and PFTBA enable PNPs to exert a superior photodynamic effect compared to ordinary photosensitizers. Based on the unique affinities between O_2_/^1^O_2_ and PFTBA, with irradiation, the PNPs can efficiently convert tumour O_2_ into cytotoxic ^1^O_2_, resulting in oxygen consumption, vascular perforation, tissue necrosis and apoptosis for improved HBA penetration.

Second, the PNPs maintained the increased intratumoural drug permeability and hypoxia for over 24 h after laser irradiation, which is long enough to allow both the delivery and activation of HBAs. This function is achieved by the PFTBA rather than the photodynamic treatment. The oxygen capacity of PFTBA is 20 times higher than that of water, which enables the PNPs to serve as buffers to restrict tumour oxygen recovery. Pimonidazole and HIF-1α staining showed that PNP-treated tumours were more hypoxic than tumours in the control groups even at 24 h after irradiation. In addition, PFTBA has an inhibitory effect on platelets^30–33^, which plays a crucial role in limiting the penetration of drugs into tumours^40–44^. Other assays revealed that intravenously injected Evans blue, TPZ and VNP20009 accumulated more in PNP-treated tumours, even without any laser stimulation.

A possible problem with using PNPs to regulate the tumour environment is the potential pro-progression effects. Enhanced hypoxia may promote tumour aggression and immune suppression and further promote tumour spreading^56^. additionally, increased tumour penetration may provide more opportunities for tumour spreading^57^. However, at the same time, recent studies have reported that the pro-progression effects of pre-hypoxia treatments could be reversed by HBAs^58,59^. HBAs may suppress hypoxia-stimulated tumour metastasis and down-regulate the expression of Snail transcription factors, which are responsible for cancer invasion and metastasis^60^. In addition, increased tumour penetration may enhance the infiltration and response of immune cells^61^. In conclusion, the advantages and disadvantages of using PNPs to regulate the tumour microenvironment need to be further verified. Additionally, it should be emphasized that particle diameter is a critical aspect of extravascular penetration. Although we achieved therapeutic efficacy against CT26 solid tumours with 160 nm PNPs, it is possible to achieve better results by adjusting the particle size to adapt to tumours of different types and stages^16^.

Another issue worth discussing is the potential challenge of light source in treating non-superficial diseases^62^. Currently, many technical solutions have been reported to solve this problem, and some good progress has been made. For example, using invasive fibre optics for internal irradiation^63^, using upconversion nanoparticles^64^ and triplet fusion upconversion^65^ and so on to convert penetrating NIR light into photodynamic visible light in situ. It is worth expecting that new PDT with additional device will be used for non-superficial diseases soon.

In summary, we successfully constructed a biocompatible nanotherapeutic that can specifically accumulate in tumours and simultaneously increase the tumour permeability and hypoxia to activate hypoxia-based therapies such as bioreductive and bacterial cancer therapies. Considering that the PNPs were formed by combining clinically approved blood substitutes and photosensitizers, these PNPs have significant potential for clinical translation in combination with HBAs for the treatment of solid tumours.

## Methods

### Materials and reagents

HSA solution was purchased from CSL Behring AG. Olive oil was obtained from Aladdin Industrial Corporation. PFTBA was supplied by Meryer Chemical Technology Co., Ltd. (Shanghai, China). HIF-1α antibody was obtained from Thermal Fisher (MA5-16009), Pimonidazole (Hypoxyprobe-1 Plus Kit, Hypoxyprobe Inc., USA) and its antibody (clone 4.3.11.3; FITC-MAb1), IR780, calcein-AM and propidium iodide (PI) were provided by Sigma-Aldrich Chemical Corporation. Alamar Blue cell viability reagent was supplied by Thermo Fisher. Singlet Oxygen Sensor Green and carbon-H_2_DCFDA were obtained from Molecular Probes. The O_2_ Consumption Rate Assay Kit was supplied by Cayman Chemical Company, and the Hypoxia/Oxidative Stress Detection Kit was purchased from Enzo. Cell Counting Kit 8 was supplied by Dojindo Laboratories (Japan). For western blot detection of HIF-1α, anti-HIF-1α antibody was obtained from Abcam (ab51608); anti-β-actin antibody was supplied by Seville (GB13001-1). For ex vivo immunofluorescence staining, anti-Salmonella antibody (FITC) was purchased from Abcam (ab69253), Glut-1 polyclonal antibody was obtained from Proteintech (21829-1-AP) and anti-CD31 antibody was provided by BioLegend (102508). The mouse CT26 colorectal cancer cells and 4T1 breast cancer cells were purchased from China Type Culture Collection obtained from the American Type Culture Collectio. BALB/c mice were purchased from the Yangzhou University Medical Centre (Yangzhou, China); the mice weighed ~20–25 g.

### Synthesis of NPs, ONPs and PNPs

NPs were formed according to protocols described below. Briefly, 120 mg of HSA was added to 60 mL of deionized water and stirred for 10 min. Then, IR780, which was dissolved in ethanol, was slowly added to the HSA solution to form NPs. To remove free IR780, the NPs were hyper-filtrated by a 30 kDa Millipore filter. Centrifugation (1438 × *g*, 5 min) was used to remove large aggregates of IR780 post ultrafiltration.

To prepare ONPs and PNPs, an unfolding/self-assembling method was adopted^36^. Briefly, 100 mg of HSA was mixed with 3 mL of deionized water and stirred for 5 min. Specified quantities of IR780, PFTBA or oil were added to the HSA solution. ONPs and PNPs were formed under sonication at 300 W in an ice bath for 10 min. Free IR780 was removed by ultrafiltration in centrifuge tubes (Millipore) for 10 min.

### Characterization of NPs, ONPs and PNPs

DLS (90Plus, Brookhaven Instruments Corp.) was employed to measure the particles size. The structure of the different samples was characterized by TEM (JEM-2100, Japan). To measure the amount of IR780, the NPs were dissolved in DMSO and then detected by UV–vis absorption spectra (UV-2450, Shimadzu, Japan).

### In vitro O_2_ absorption and consumption

An O_2_ microelectrode (Unisense) was used to explore the hypoxic conditions mediated by PNPs in vitro. Briefly, 1 mL of water was added to a vial and covered with mineral oil for to separate it from air. Then, 0.06 mL of PNPs was mixed into the water, with a final IR780 concentration of 2 µg mL^−1^ (1% PFTBA, v/v) and 4 µg mL^−1^ (2% v/v PFTBA). DO was monitored using the microelectrode before and after 10 min of laser irradiation (808 nm, 400 mW cm^−2^).

To evaluate O_2_ absorption, the sample (8 µg mL^−1^ IR780, 4% v/v PFTBA) was deoxygenated (N_2_ flow rate = 0.01 L min^−1^ for 30 s, step 1) and then (step 2) irradiated at the same intensity as above with O_2_ microelectrode detection. Repeated O_2_ consumption profiles were measured using the same method, except the samples were alternately exposed to irradiation or kept in the dark every 10 min. The irradiated PNPs were then combined with air-saturated water as an O_2_ supply before the next exposure.

### Hypoxia assessment using an O_2_ microelectrode in vivo

To detect the PNP-mediated O_2_ absorption in vivo, 50 µL of deoxygenated PNPs (IR780, 1.4 mg kg^−1^; 20% v/v PFTBA) were injected into the tumour. Dissolved O_2_ was monitored using the microelectrode before and after the injection. To measure laser-triggered O_2_ alterations, the tumour was subjected to laser irradiation (808 nm, 2 W cm^−2^) for 20 s immediately after the intratumoural injection of 50 μL of PNPs (1.4 mg kg^−1^ IR780, 20% v/v PFTBA). These mice were exposed to the laser three times.

Then, low-intensity laser irradiation (808 nm, 400 mW cm^−2^) was used to increase the tumour hypoxia in vivo. At 24 h after the injection of 200 μL of PNPs (1.4 mg kg^−1^ IR780, 20% v/v PFC), the mice were irradiated (808 nm, 400 mW cm^−2^) for 5 min. The O_2_ microelectrode was placed ~1 mm into the tumour to monitor the changes in O_2_.

### Ex vivo immunofluorescence staining

Ex vivo immunofluorescence staining of the hypoxyprobe (pimonidazole hydrochloride) and HIF-1α were employed to evaluate the hypoxic conditions in the tumours. Briefly, 200 µL of the samples (1.4 mg kg^−1^ IR780; 20% v/v PFTBA) was intravenously injected into the CT26 tumour-bearing mice; the mice were then irradiated (808 nm, 400 mW cm^−2^) for 5 min at 24 h post injection. The mice then received an injection of pimonidazole hydrochloride intravenously at a dose of 60 mg kg^−1^. Tumour sections were sequentially stained for HSA, treated with Triton X-100 and stained with anti-pimonidazole antibody (1:200, clone 4.3.11.3; FITC-MAb1). Finally, the sections were imaged using a digital microscope (Nikon, Japan). To measure the expression of HIF-1α, treatments similar to pimonidazole staining were performed, and the mice were sacrificed 24 h later. The tumours were removed and treated according to the instructions of the HIF-1α Immunocytochemistry Kit (anti-HIF-1α antibody was diluted with 1:200). Deoxygenated PNPs (N_2_ flow rate = 0.01 L min^−1^ for 1 min) were intratumourally injected into mice for this evaluation of HIF-1α expression. Images of the HIF-1α expression in tumour sections were captured by microscopy. The hypoxia status, as indicated by the staining intensity of each section, was statistically analysed using the ImageJ software.

### Assessment of PNP-induced Evans blue penetration in vivo

Evans blue was employed to evaluate changes in tumour penetration mediated by PNPs under laser irradiation. Briefly, 200 µL of the samples (PNPs [1.4 mg kg^−1^ IR780; 20% v/v PFTBA], ONPs, NPs or saline) were injected intravenously into the mice when the CT26 tumour diameter reached 6–8 mm. Then, the tumours were irradiated (808 nm, 400 mW cm^−2^) for 5 min at 24 h post administration. Evans blue dye (20 mg kg^−1^ in 0.1 mL of PBS) was also injected intravenously 1 h before laser irradiation; 3 h later, the mice were sacrificed, and the tumours were removed and weighed. Evans blue dye in the tumour was extracted with formamide and quantified using a UV–vis spectrometer (optical density = 620 nm). Ex vivo images of tumour sections stained with Evan blue were also obtained after the same treatments. To explore PNP-mediated changes in tumour permeability without laser irradiation, similar treatments were performed without laser irradiation.

### Western blot analysis of HIF-1α protein in tumour

Tumour-bearing mice injected with different samples intravenously were exposed laser (808 nm, 400 mW cm^−2^) for 5 min, and then those tumours were washed with ice-cold PBS and ground in 4 mL extract buffer on ice for 30 min. Using a micro-centrifuge, the extract was spun down at a rate of 10,000 × *g* for 5 min at 4 °C, and the lysate was acquired for further analysis of corresponding proteins. Then, proteins were resolved using sodium dodecyl sulphate-polyacrylamide gel electrophoresis and transferred onto a PVDF membrane. The membrane was then incubated with a dilute solution (1:1000) of anti-HIF-1α and anti-actin antibody, which was used to detect the actin proteins as a loading control for whole-cell lysate after blocking with 0.05% Tween-20 and 5% skimmed milk in PBS. All the antibodies were purchased from Wuhan Servicebio Technology Co., Ltd. The results of western blot were performed to visualize using the chemiluminescence technology. Unprocessed scans of the most important blots were supplied in the Source Data file. In addition, corresponding quantifications were measured by the Alpha software.

### Animal model

Five-week-old BALB/c male mice weighing ~20–22 g were purchased from the Yangzhou University Medical Centre (Yangzhou, China), and the animals were cared for and used in accordance with the regulations of the Institutional Animal Care and Use Committee (IACUC) of Nanjing University. During the study, these animals had free access to food and water. Before treatment, the hair on the flanks of the mice was removed; then, anaesthesia was induced with an intraperitoneal injection of 1% pentobarbital sodium (100 µL) anaesthesia. To establish the subcutaneous tumour models, 1 × 10^7^ CT26 cells suspended in 100 µL of serum-free Dulbecco's modified Eagle's medium were injected into the lower flanks of the mice subcutaneously. When the tumour volume reached ~150 mm^3^, the tumour mass was cut into smaller pieces with a volume of 2–5 mm^3^, which were then subcutaneously implanted into other mice.

During the study, animals were observed every day for any clinically relevant abnormalities. If any mouse was moribund due to treatment-induced toxicity, the appearance of severe ulceration around the ectopic tumour or the loss of 20% of body weight compared with the start of study before scheduled killing, it was euthanized. Mice were treated in accordance with NJU-IACUC-approved animal use guidelines.

### PNPs enhanced TPZ chemotherapy by the intravenous injection of TPZ

Mice (20–25 g) bearing CT26 tumours (60–80 mm^3^) were randomly divided into six groups (*n* ≥ 6). The treatment scheme was as follows: 1. saline; 2. TPZ; 3. ONPs; 4. PNPs; 5. ONPs plus TPZ; and 6. PNPs plus TPZ. Both PNPs (1.4 mg kg^−1^ IR780; 20% v/v PFTBA) and TPZ (20 mg kg^−1^) were injected intravenously into mice on days 1, 3 and 5. Tumours in groups 3, 4, 5 and 6 were irradiated three times (808 nm, 400 mW cm^−2^) for 5 min at 24 h after the administration of the PNPs. Then, the tumour length (*L*) and width (*W*) were recorded every day using a digital calliper, and the volume (*V*) was calculated according to the following formula: *V* = *L* × *W*^2^/2. Mice in each group were sacrificed on day 13, and the tumours were isolated and weighed. The therapeutic efficacy of the different treatments was also evaluated by H&E and TUNEL staining on day 8.

### Haematologic indexes and blood biochemistry

CT26 tumour-bearing mice (20–25 g, *n* = 3) were used in this study. When the tumour size reached 60–100 mm^3^, VNP20009 (5 × 10^7^ CFU mL^−1^, 100 µL) or PBS was intravenously injected into the mice. At different time points (1 and 7 days), blood and serum samples were obtained. Haematologic indexes were measured using an autohaematology analyser (Sysmex XE2100), and blood biochemical parameters were evaluated using corresponding kits according to their instructions.

### VNP20009 distribution in CT26 tumour-bearing mice

The VNP20009 strain was transferred to a fresh LB medium. After the OD value reached 0.8, the VNP20009-containing medium was centrifuged at 3500 × *g* at 4 °C. Then, VNP20009 was collected and washed twice with sterile PBS and quantified by UV–vis spectroscopy (OD = 600 nm). A total of nine mice bearing CT26 tumours were divided into three groups. The mice received 200 µL of PNPs (1.4 mg kg^−1^ IR780; 20% v/v PFTBA), ONPs or saline intravenously. VNP20009 (5 × 10^6^ CFU mouse^−1^) was intravenously injected into the mice 1 h before laser irradiation. All tumours were irradiated (808 nm, 400 mW cm^−2^) for 5 min at 24 h after the injection of VNP20009. The mice were sacrificed 3 days after irradiation, and the heart, liver, spleen, lungs, kidneys and tumour were removed under sterile conditions. After being weighed, these tissues were homogenized in pre-cooled PBS, diluted and coated onto LB plates. The number of VNP20009 colonies was calculated after 12–16 h of culture. After similar tumour treatments, ex vivo immunofluorescence analysis was also performed for glut-1 and Sa. Anti-glut-1 and anti-Sa. antibodies were diluted with 1:200.

### PNPs enhanced bacterial cancer therapy

To explore the enhancement of bacterial cancer therapy mediated by the PNPs, male BALB/c mice bearing CT26 tumours 60–80 mm^3^ in size were randomly divided into six groups. The treatment scheme was as follows: 1. saline; 2. PNPs; 3. ONPs; 4. Sa.; 5. ONPs plus Sa.; and 6. PNPs plus Sa. The mice were treated and sacrificed on day 10; then, the tumours in each group were weighed.

### Statistical analysis

Statistical analysis was performed using two-tailed Student’s *t* test for two groups and one-way analysis of variance for multiple groups. *P* values: *p < 0.05, **p < 0.01, ***p *<* 0.001 (unpaired, two-way *t* tests). *P* values < 0.05 were considered statistically significant.

### Reporting summary

Further information on experimental design is available in the Nature Research Reporting Summary linked to this article.

## Supplementary information

Supplementary Information

Peer Review File

Reporting Summary

Source Data

## Data Availability

The authors declare that the main data that support the findings of this study are available from the corresponding author upon reasonable request. A reporting summary for this study is available as a Supplementary Information file. The source data underlying Figs. 1c, 2a, 2c–d, 2f, 3a, 3c–e, 3i, 4b–f and 5b–f and Supplementary Fig. 1, 2a–b, 4b–c, 5–7, 9, 11–14, 16–17, 18a, 18c, 20, 21a–c, 26 and 28 are provided in a source data file.
