# Supplementary material for: Perfluorocarbon regulates the intratumoural environment to enhance hypoxia-based agent efficacy
Source: Nat Commun. 2019 Apr 5;10:1580. doi: 10.1038/s41467-019-09389-2 (PMC6450981; doi:10.1038/s41467-019-09389-2)
Supplement: Supplementary file 1 — Supplementary Information [file 41467_2019_9389_MOESM1_ESM.pdf]

Supplementary Information

Perfluorocarbon regulates the intratumoural environment to enhance hypoxia-based agent efficacy

Wenguang Wang[1,2,†], Yuhao Cheng[1,2,†], Peng Yu[1,2,†], Haoran Wang[1,2], Yue Zhang[1,2], Haiheng Xu[1,2], Qingsong Ye[1,2], Ahu Yuan[1,2,*], Yiqiao Hu[1,2,3,*], Jinhui Wu[1,2,3,*]

Affiliations:

[1] State Key Laboratory of Pharmaceutical Biotechnology, Medical School of Nanjing University & School of Life Sciences, Nanjing University, Nanjing 210093, China

[2] Institute of Drug R&D, Nanjing University, Nanjing 210093, China

[3] Jiangsu Provincial Key Laboratory for Nano Technology, Nanjing University, Nanjing 210093, China

[†] These authors contributed equally to this work.

[*] **Corresponding authors:**

Jinhui Wu, Ph.D. (Main corresponding author)

Email: wuj@nju.edu.cn

Yiqiao Hu, Ph.D.

Email: huyiqiao@nju.edu.cn

Ahu Yuan, Ph.D.

Email: yuannju@nju.edu.cn.

Phone: +86-25-83596143

Address: 22 Hankou Road, Nanjing University, Nanjing 210093, China

Supplementary Figures

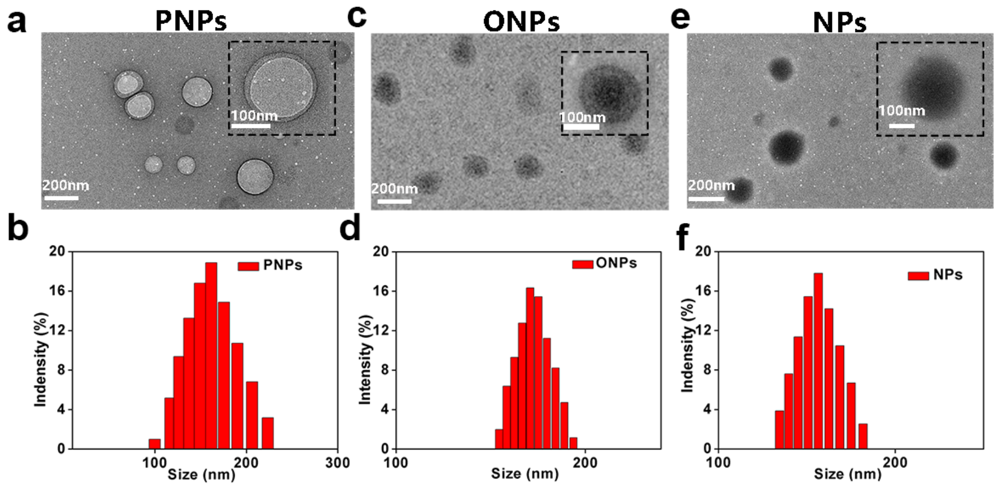

Supplementary Fig. 1 The morphology of PNPs (a), ONPs (c) and NPs (e) observed by transmission electron microscopy and their respectively size distributions determined by dynamic light scattering (b, d, f).

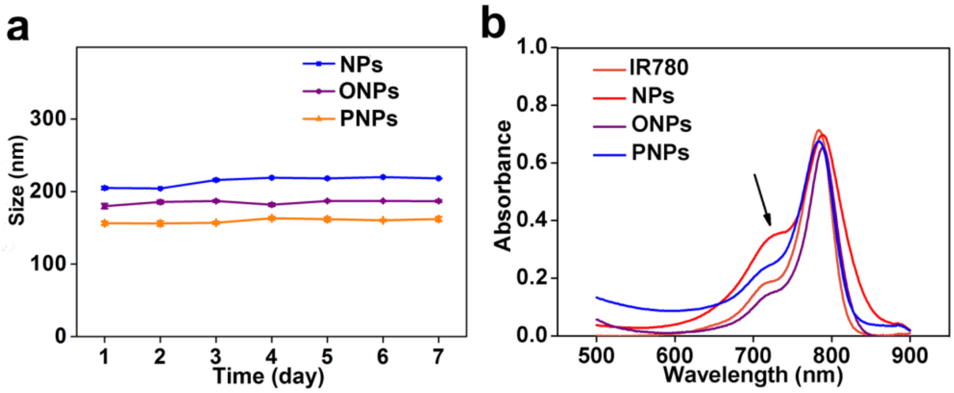

Supplementary Fig. 2 (a) Stability of NPs, ONPs and PNPs in PBS at 4 °C. Values are the mean ± s.d. (*n*=3) (b) UV-vis spectra of free IR780, NPs, ONPs and PNPs. The black arrow represents the π-π stacking shoulder peak of IR780 in NPs.

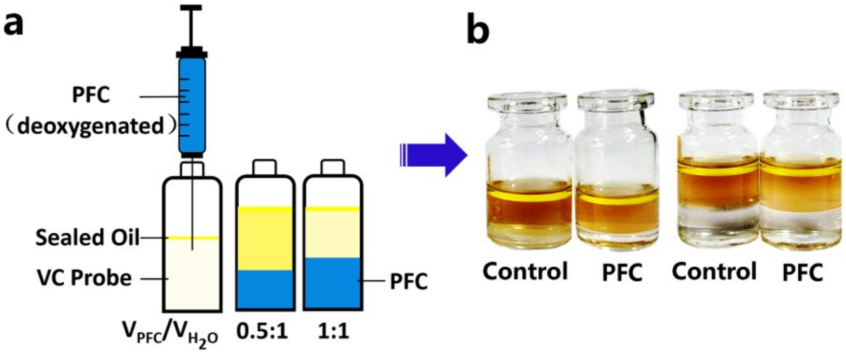

Supplementary Fig. 3 Deoxygenated PFTBA mediated O_2 absorption. (a) Schematic diagram of O_2 absorption reflected by the oxidation of vitamin c. Deoxygenated PNPs were added into H_2O and sealed with oil. (b) The images of vitamin c after deoxygenated PFTBA added. Oxygen concentration was reflected by the brownish color of oxidative vitamin c. The darker the color, the more oxygen.

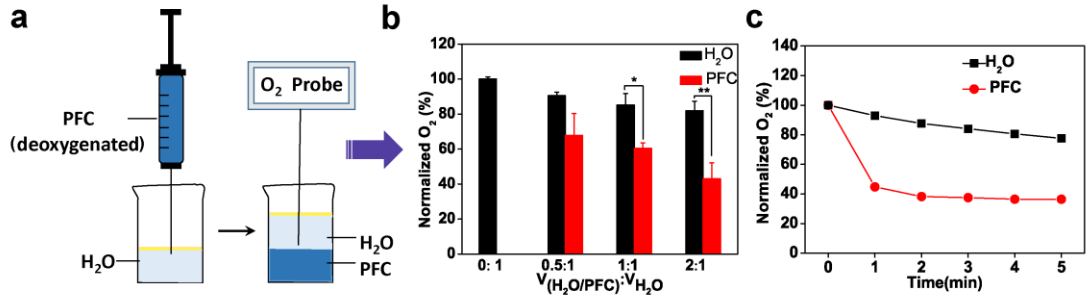

Supplementary Fig. 4 Deoxygenated PFTBA mediated O_2 absorption. (a) Schematic diagram of O_2 changes in water. (b) Normalized decrement of O_2 by deoxygenated PFTBA further confirmed the good O_2 absorption of PFTBA. Values are the mean ± s.d. (*n*=3). (c) Time dependent O_2 changes monitored by O_2 meter.

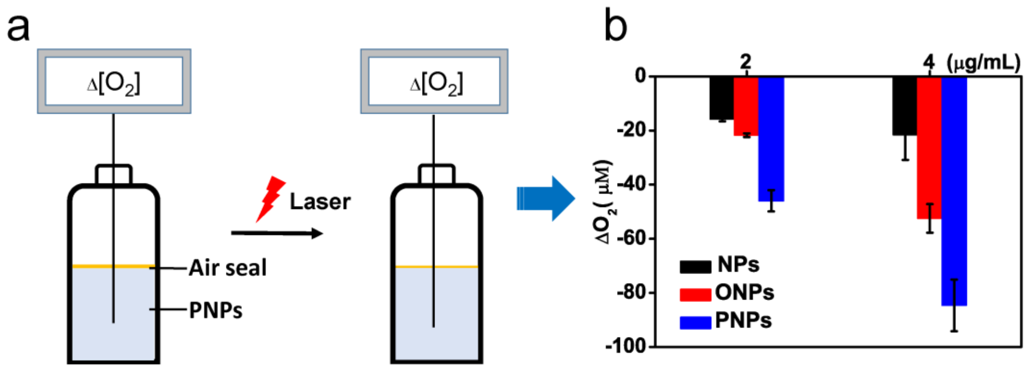

Supplementary Fig. 5 (a) The DO (dissolved O_2) measuring process in water post laser irradiation. PNPs were mixed into H_2O and sealed with oil. After laser irradiation (808 nm, 400 mW cm^{-2}) for 10 min, the DO was measured by O_2 meter; (b) Quantification of DO in water. The change of DO was achieved by the records before and after laser irradiation. The largest O_2 change was achieved in PNPs group. Values are the mean ± s.d. (*n*=3).

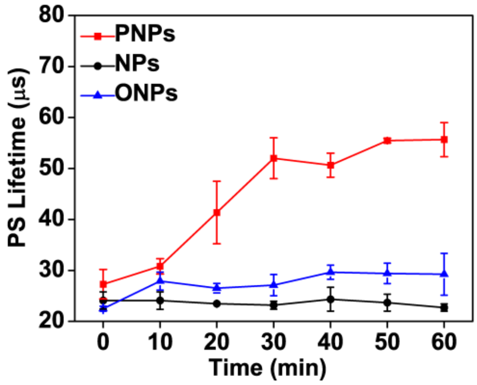

Supplementary Fig. 6 Phosphorescence (PS) lifetime profile of phosphorescent molecular probe over laser irradiation. The lower PO_2, the longer the PS lifetime. The fact that PNPs produced longer PS lifetime indicated that a more hypoxic environment could be achieved by PNPs. Values are the means ± s.e.m. (*n*=2).

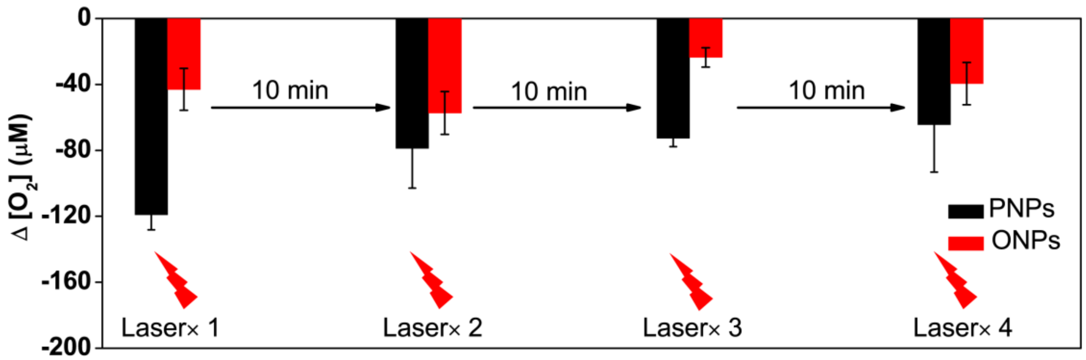

Supplementary Fig. 7 Dissolved oxygen variations (Δ [O_2]) of PNPs treated by repeated light/dark cycles. Values are the means ± s.d. (*n*= 3).

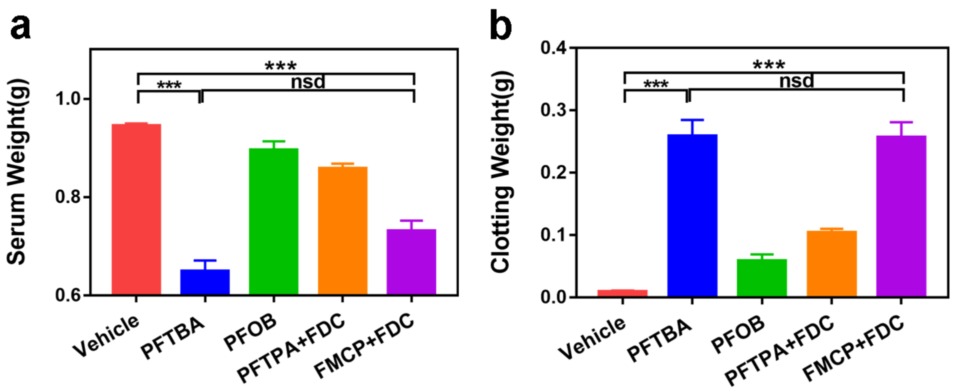

Supplementary Fig. 8 The platelet inhibition was evaluated by blood clot retraction. Both serum weight (a) and clotting weight (b) were measured after in vitro incubated with platelet which was pretreated with perfluocarbons (PFCs) (PFOB: perfluorooctylbromide; PFTPA: perfluorotripropylamine; FDC: perfluorodecalin; FMCP: perfluorocyclohexyl piperidine). Result suggested that PFTBA could inhibit platelets most effectively among those PFCs ($n \geq 4$). Values are the mean ± s.d. nsd: no significant difference, *** $p < 0.001$ (unpaired, two-way t tests).

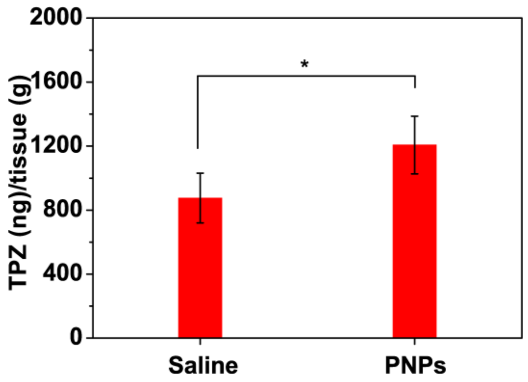

Supplementary Fig. 9 The distribution of TPZ in tumour of CT26 bearing mice. TPZ was administrated intravenously at 5 h after PNPs were i.v. injected into mice. At 10 h post intravenous administration of TPZ, mice were sacrificed and the level of TPZ in different tissues was measured by high performance liquid chromatography (HPLC). Values are the mean ± s.d. (saline, *n*= 4; PNPs, *n*=5) (unpaired, two-way *t* tests).

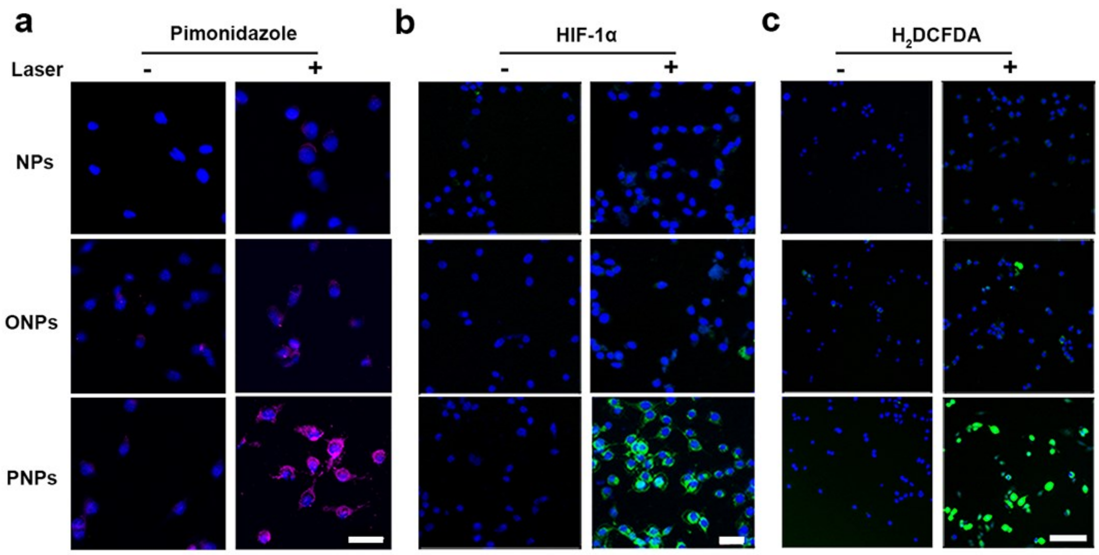

Supplementary Fig. 10 Cellular $^1O_2$ and hypoxia assays by PNPs and irradiation. (a) Fluorescence images of hypoxia (a), HIF-1α (b) and $^1O_2$ (c) in CT26 cells. Cells were respectively detected by Hypoxia stress detection kit (purple; scale bar, 25 μm), HIF-1α antibody (green; scale bar, 50 μm) and H_2DCFDA (green; scale bar, 100 μm) respectively. Nuclei were stained by DAPI (blue).

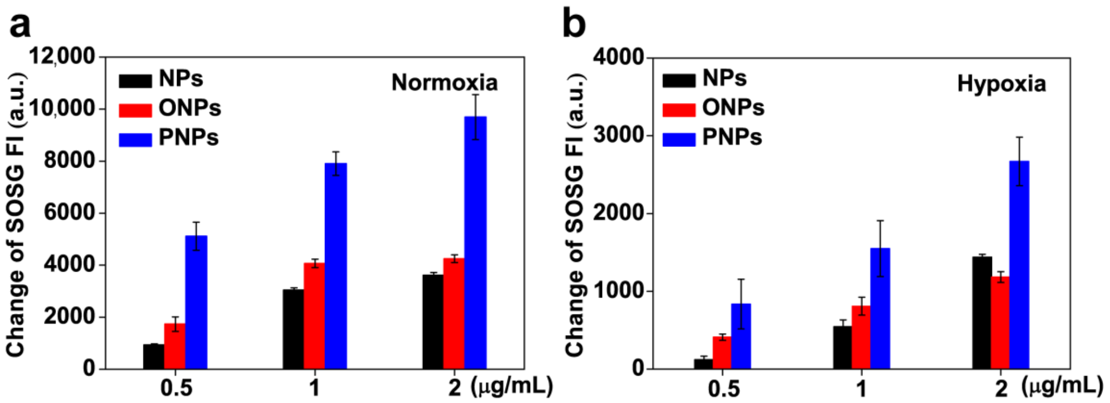

Supplementary Fig. 11 Photodynamic efficacies of PNPs. (a-b) Laser stimulated $^1O_2$ generation under normoxic (21% O_2) and hypoxic (1% O_2) conditions, as determined by the fluorescence intensity of SOSG ($^1O_2$ probe, *n*=3).

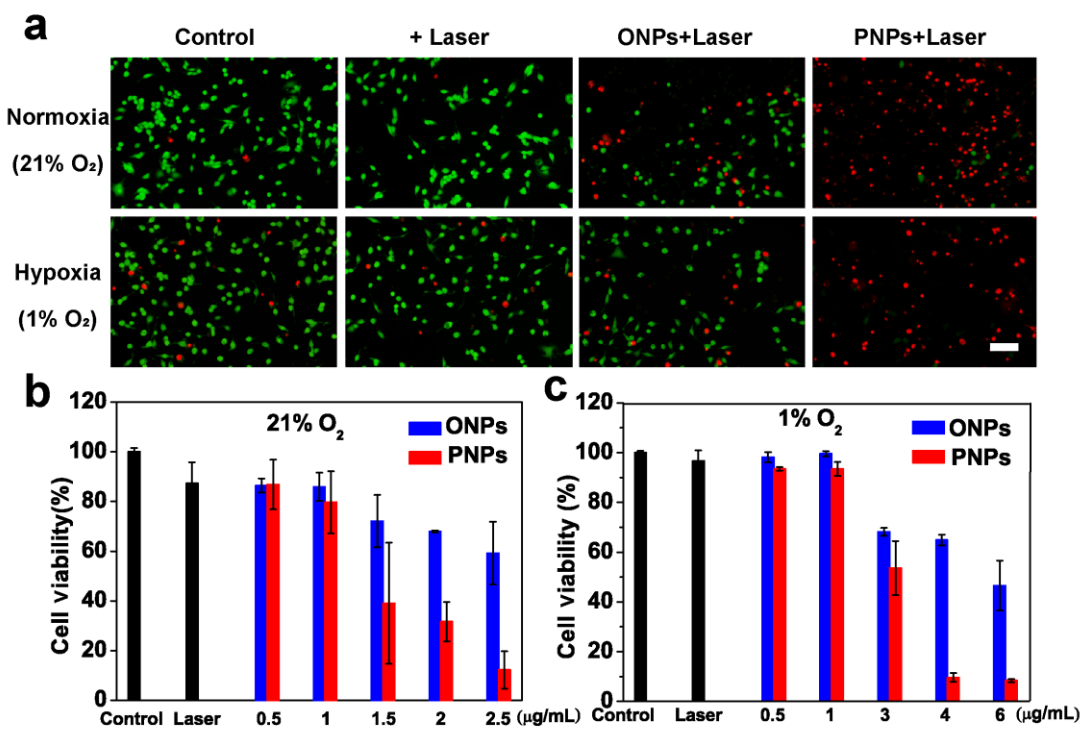

Supplementary Fig. 12 Enhanced cytotoxicity by PNPs mediated PDT in normoxic and hypoxic conditions. Cells were exposed to intermittent irradiation (808 nm, 400 mW cm^{-2}) for 30 s x 2 times, and viability was measured by AlarmBlue assay and calcein-AM/propidium iodide (PI) staining. (a) Live/dead staining of CT26 cells treated with ONPs or PNPs (live cells, green; dead cells, red). Scale bar: 100 μm. (b) Cell viability of treated CT26 cells in normoxic condition (21% kPa O_2) and (c) in hypoxic condition (1% kPa O_2). Values are the mean ± s.d (n=3).

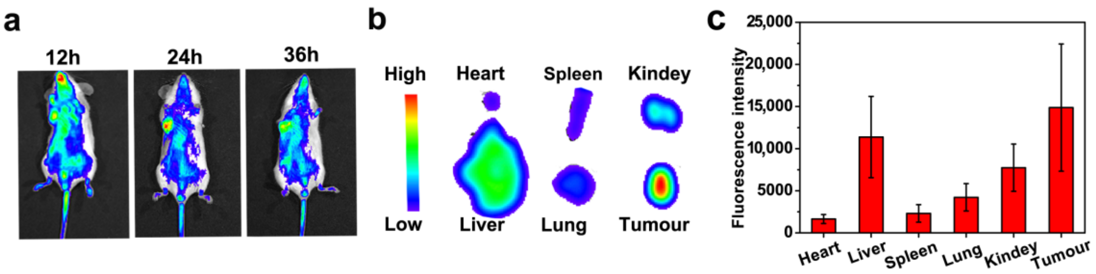

Supplementary Fig. 13 Bio-distribution of PNPs. (a) Near-infrared imaging of tumour accumulation of IR780 (PNPs) in CT26-bearing mice after intravenous injection of PNPs. Images were taken at 12, 24 and 36 h post injection. (b) Bio-distribution of PNPs in mice determined by the IR780 fluorescence from tissue at 24 h post injection. (c) Semi-quantitative analysis of IR780 in tumor and tissue after intravenous injection of PNPs. IR780 was determined by NIR fluorescence. Values are the mean ± s.e.m. (*n*=4).

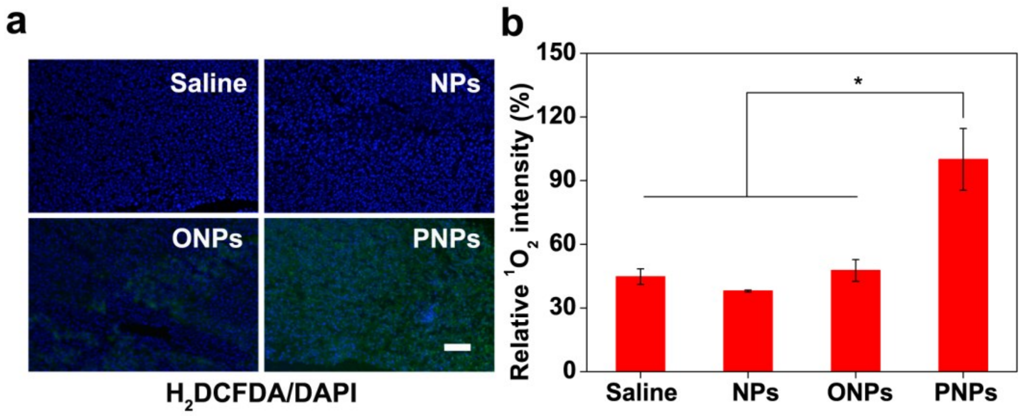

Supplementary Fig. 14 (a) Ex vivo Immunofluorescence staining of $^{1}O_2$ detected by H_2DCFDA (green, scale bar, 200 μm) and their corresponding quantification (b). Values are the mean ± s.e.m. (Saline, *n*=3; NPs, *n*=3; PNPs, *n*=5; ONPs, *n*=6) (unpaired, two-way *t* tests).

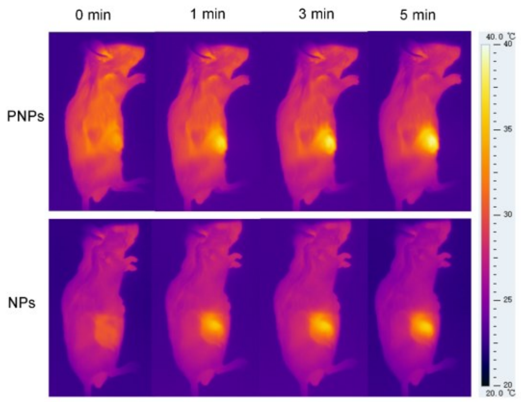

Supplementary Fig. 15 IR thermal images of CT26-tumour-bearing mice injected with PNPs and NPs under the 808 nm laser (400 mW cm^{-2} for 5 min) irradiation.

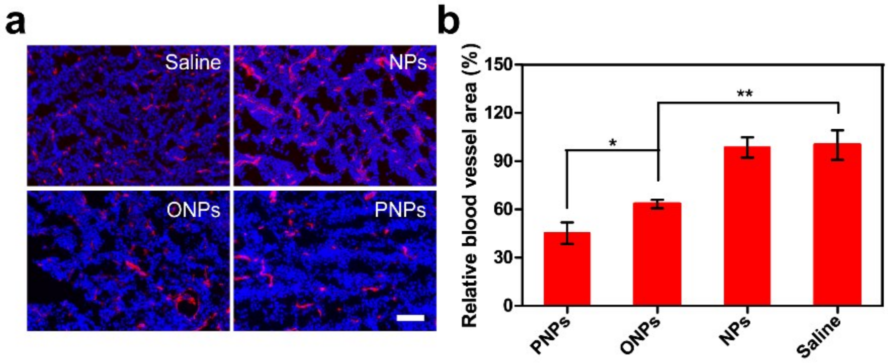

Supplementary Fig. 16 Ex vivo immunofluorescence images of tumour vessels post laser irradiation. (a) The tumour vessels and nuclei were stained with anti-CD31 antibody (red) and DAPI (blue), respectively. (b) Quantification of blood vessel densities after irradiation. Values are the mean ± s.e.m. (Saline, PNPs, NPs, *n*=5; ONPs, *n*=6) $*p < 0.05$, $**p < 0.01$ (unpaired, two-way *t* tests).

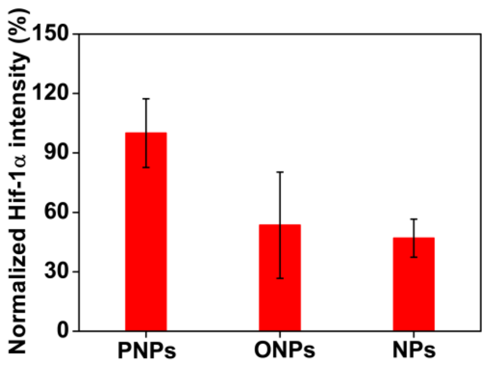

Supplementary Fig. 17 The corresponding quantitative levels of HIF-1α protein (western blot) by the different treatments. The quantitative results were calculated by protein expressions/actin HIF-1α (For PNPs and NPs, *n*=3; For ONPs, *n*=2). Values are the mean ± s.e.m.

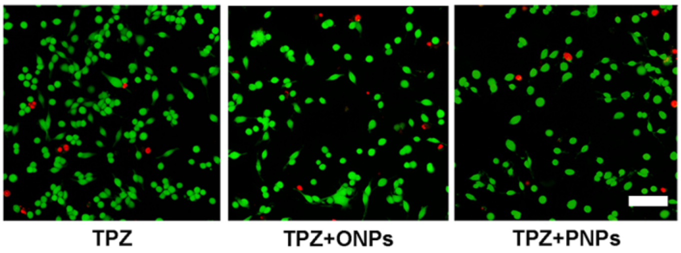

Supplementary Fig. 18 Fluorescence images of CT26 cells cultured with TPZ and unoxygenated ONPs or PNPs. Viable cells were stained by calcein-AM (green), and dead/later apoptosis cells were stained by PI (red, scale bar: 100 μm).

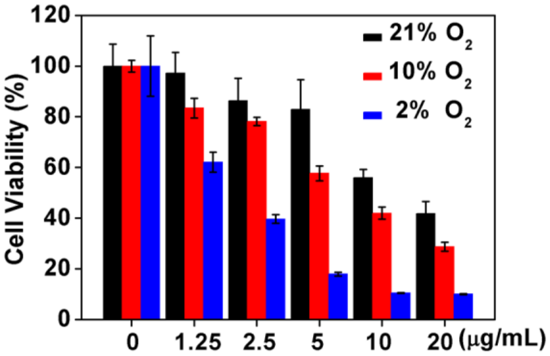

Supplementary Fig. 19 Cytotoxicity of treated CT26 cells after co-incubation with TPZ for 24 h under different O_2 environments, including 21% kPa O_2 (normoxia), 10% kPa O_2 (semi-hypoxia) and 2% kPa O_2 (hypoxia), measured by Cell Counting Kit-8 method in vitro. Values are the mean ± s.d. (*n*=6).

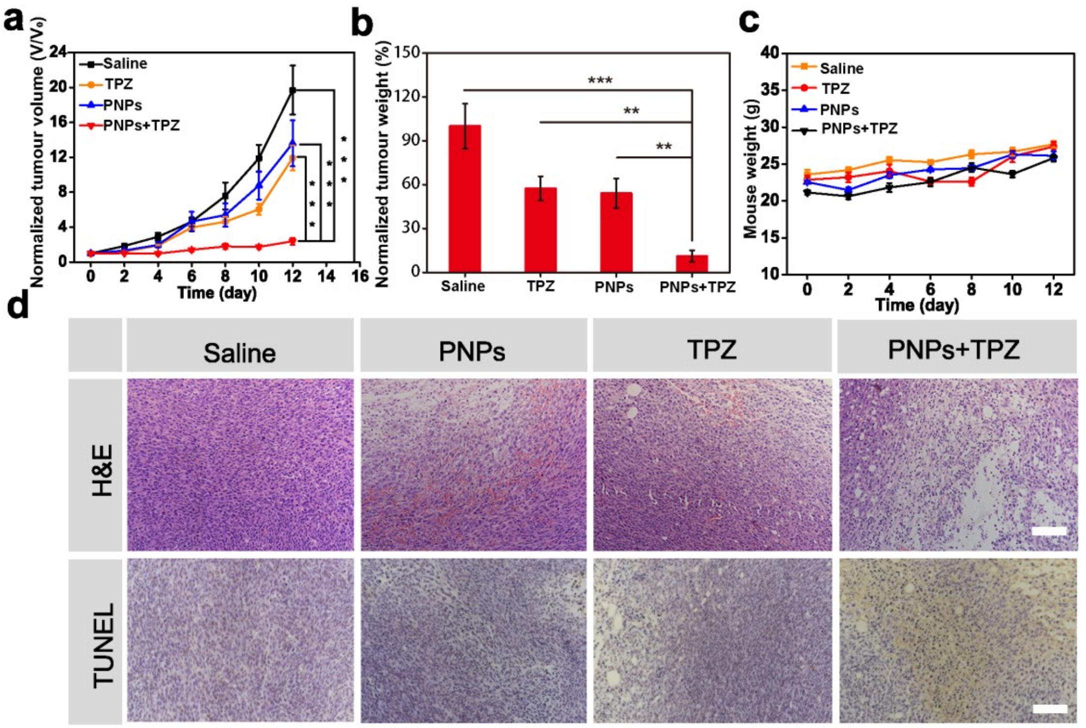

Supplementary Fig. 20 PNPs enhanced TPZ chemotherapy. (a) Tumour growth curves of treated mice. (*n*= 5-7 mice per group). (b) Normalized averages of tumour weights at day 12. (c) Changes of body weight. (d) H&E and TUNEL stained CT26 slices. Samples were collected from different groups at day 2 post administration (scale bars, 100 μm). Values are the mean ± s.e.m. $^{*}p < 0.05$, $^{**}p < 0.01$, $^{***}p < 0.001$ (unpaired, two-way *t* tests).

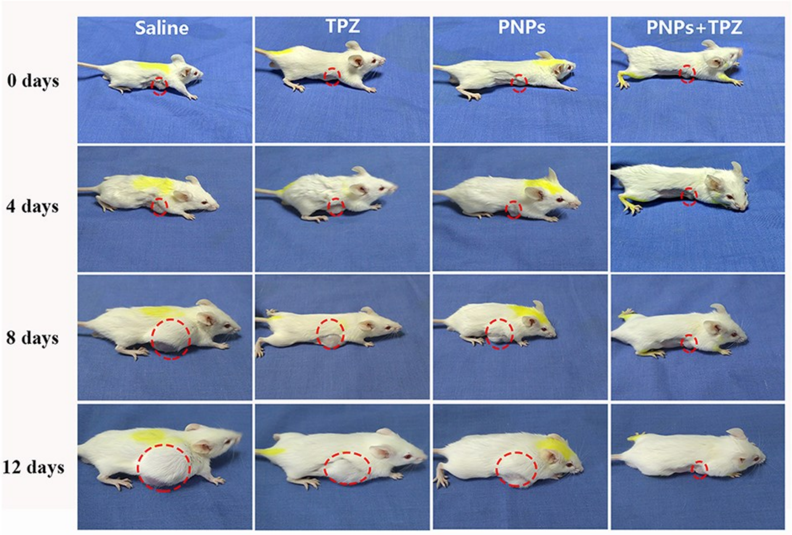

Supplementary Fig. 21 Photographs of mice from different groups after corresponding treatments, including control (saline), free TPZ, PNPs and TPZ plus PNPs. All of the groups were exposed to laser (808 nm, 400 mW cm^{-2}) for 5 min at 24 h after intravenous administration.

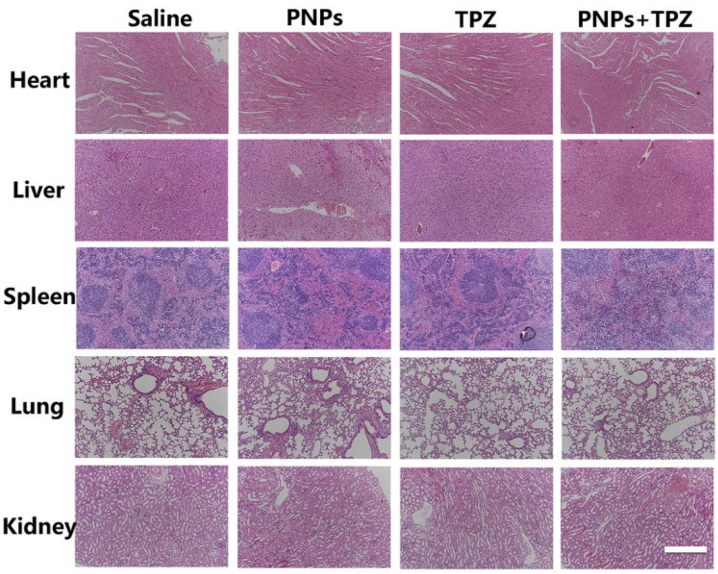

Supplementary Fig. 22 Histological analysis of the organs acquired from CT26 bearing mice on day 12 post-injection with saline, PNPs, TPZ and PNPs plus TPZ (scale bars, 100 μm).

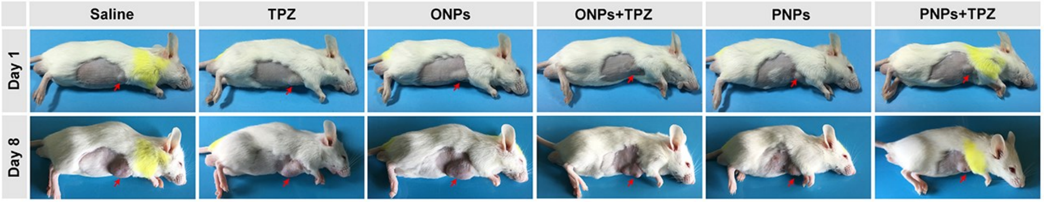

Supplementary Fig. 23 Photographs of mice from different groups after corresponding treatments, including control (saline), free TPZ, ONPs, PNPs, TPZ plus ONPs and TPZ plus PNPs. TPZ (20 mg kg^{-1}) was intravenously injected into mice 3 h before laser irradiation. All of the groups were exposed to laser (808 nm, 400 mW cm^{-2}) for 5 min at 24 h after intravenous administration of PNPs (200 μL PNPs, (1.4 mg kg^{-1} IR780), 20% v/v PFTBA).

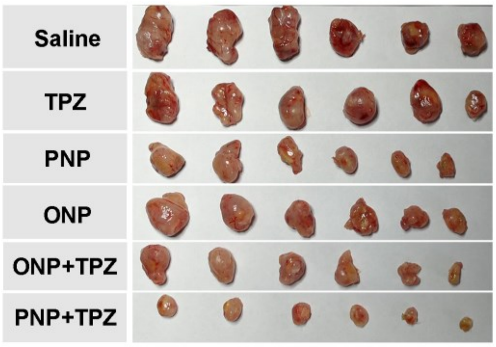

Supplementary Fig. 24 Images of tumours after treatments with different samples. Treatments as follows: saline, TPZ, ONPs, ONPs plus TPZ, PNPs, PNPs plus TPZ. TPZ (20 mg kg^{-1}) was intravenously injected into mice 3 h before laser irradiation. All of the groups were exposed to laser (808 nm, 400 mW cm^{-2}) for 5 min at 24 h after intravenous administration of PNPs (200 µL PNPs, (1.4 mg kg^{-1} IR780), 20% v/v PFTBA).

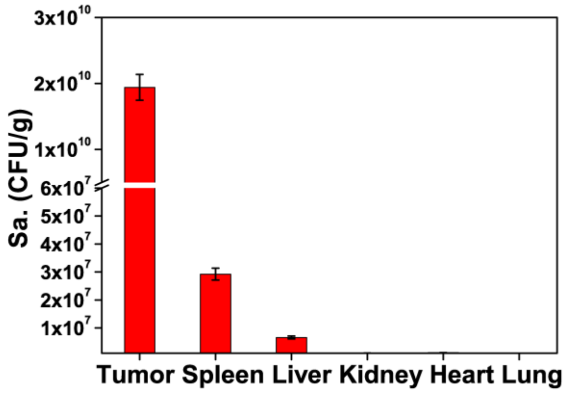

Supplementary Fig. 25 In vivo distribution of VNP20009. Tissue distributions of VNP20009 in mice which received intravenous injection with PNPs were measured by colony forming assay. More VNP20009 accumulated in tumours after laser irradiation which were intravenously injected with PNPs, almost 1000 times than their distribution in spleen. Values are the mean ± s.e.m. (*n*=3).

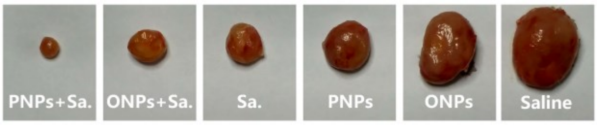

Supplementary Fig. 26 Images of tumours after treatments with different samples. Treatments as follows: saline, Sa., ONPs, ONPs plus Sa., PNPs, PNPs plus Sa.. At 24 h post injection, all those tumors were irradiated with laser (808 nm, 400 mW cm^{-2}) for 5 min after intravenous injection of VNP20009 in related groups. The VNP20009 and PNPs doses were 5×10^6 CFU per mouse and 200 μL (1.4 mg kg^{-1} IR780, 20% v/v PFC) $mouse^{-1}$ in related groups respectively.

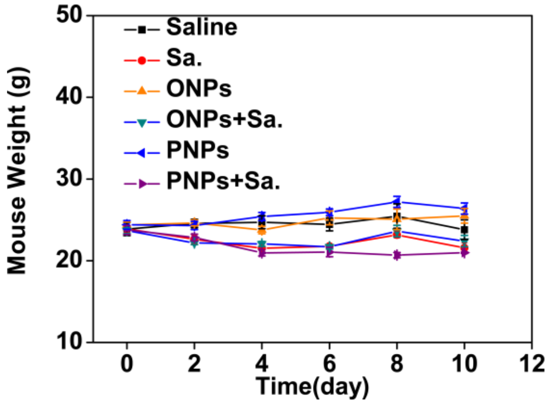

Supplementary Fig. 27 The change of body weight in different groups.

Supplementary Methods

Detection of singlet oxygen in vitro. SOSG was employed to quantify the generation of $^1O_2$ according to protocol[1]. Briefly, samples (0.1 mL) and SOSG (50 μM, 0.02 mL) were mixed in 96-well plates (Costar). After laser exposure (808 nm, 2 W cm^{-2}), the oxidized SOSG was detected using a multifunctional microplate reader by measuring the fluorescence intensity (E_x/E_m = 504/525 nm). To confirm whether the $^1O_2$ generation was related to O_2 levels, samples were kept in transparent box under different O_2 partial pressure conditions, respectively (1% and 21% kPa O_2), then oxidized SOSG with 20 s laser irradiation was also quantified by measuring the fluorescence intensity. Experiments for each group were run in triplicate.

Carboxy-H_2DCFDA was employed as a ROS indicator to monitor intracellular ROS by fluorescence microscopy (Nikon, Japan). CT26 cells were seeded with a density of 5 × 10^3 per well in 96-well plates. After the cells were incubated for 24 h, different samples with the final concentration of IR780 of 4 μg/mL were added to each well. Then, the cells were further incubated for 30 min at 37 °C and 5% CO_2. The cells were incubated with 100 μL per well carboxy-H_2DCFDA (25 mM) for 30 min after washing once with PBS. Subsequently, the cells were washed with PBS and irradiated by laser exposure (808 nm, 2 W cm^{-2}) for two consecutive 10 s exposures. An 1 minute interval was added between the two irradiations for avoiding high temperature. Then, the cells were labelled with 100 μL Hoechst 33342 (1 mM) for 5 min. The fluorescence emission spectrum of carboxy- H_2DCFDA (Ex/Em = 495/529 nm) and Hoechst 33342 (Ex/Em = 350/461 nm) were immediately performed on a fluorescence microscope (Nikon, Japan).

O_2 consumption rate assay by TRF. O_2 consumption rate was measured by MitoXpress Kit (Cayman Chemical) according to the procedure provided by the manufacturer. DPBF was added as a quencher of $^1O_2$ because the MitoXpress

probe can be oxidized by $^1O_2$ irreversibly. Briefly, 150 μL PNPs solution (8 μg mL^{-1} IR780, 4% v/v PFTBA) and 10 μL DPBF (0.1 mM) were placed in a 96-well plate, covered with mineral oil according to the manufacturer's protocol, then samples were exposed to laser (808 nm, 400 mW cm^{-2}) for 5 min. Then MitoXpress probe (10 μL) was added into the mixed solution for measurement. The 96-well plate was detected by a microplate reader with the excitation/emission wavelength of 530/585 nm. Each sample was irradiated repeatedly every 1 minute, then measured every 10 minutes, with two readings at delay times of 30 and 70 μs and a gate time of 30 μs. Acquired time-resolved fluorescence (TR-F) intensity signals were converted into the phosphorescence lifetime (microsecond) [τ] values by the formula: $\tau = (70-30)/\ln(F_1/F_2)$, where F_1 and F_2 are the TR-F signals at the delay times of 70 and 30 μs separately. O_2 concentration of the sample was reflected by the phosphorescence lifetime [τ]. For other samples, procedure was performed similarly.

Western blot analysis of HIF-1α protein in tumour. Tumour bearing mice injected with different samples intravenously were exposed laser (808 nm, 400 mW cm^{-2}) for 5 min, then those tumours were washed with ice-cold PBS and ground in 4 mL extract buffer on ice for 30 minutes. Using a micro-centrifuge, the extract was spun down at a rate of 10000 xg for 5 min at 4 °C, and the lysate was acquired for further analysis of corresponding proteins. Then, proteins were resolved using SDS-polyacrylamide gel electrophoresis and transferred onto a PVDF membrane. Membrane was then incubated with dilute solution (1:1000) of anti-HIF-1α and anti-actin antibody which was used to detect the actin proteins as loading control for whole-cell lysate after blocked with 0.05% tween 20 and 5% skimmed milk in PBS. All the antibodies were purchased from Wuhan servicebio technology Co., Ltd. The results of western blot were performed to visualize using the chemiluminescence technology. In addition, corresponding quantifications were measured by the Alpha software.

In vitro analysis of PFC acting as an O_2 sponge. Qualitative analysis: Oxidation discoloration of VC was employed to illustrate the effect of O_2

absorption by nano-PFC. Briefly, 5 mL VC solution was added to vial (final concentration, 10 μg mL^{-1}). Different volumes of PFC (CCl_4) were added after 0.1 mL NaOH (0.1 mM) was mixed. O_2 concentration in VC solution was reflected by the color of VC solution. Similarly, for quantitative analysis, different volume of deoxygenated PFTBA (H_2O) was added into three groups with the final ratios between PFTBA (H_2O) and H_2O were 0.5:1, 1:1 and 2:1 (v/v) respectively. Then the change of DO was monitored by O_2 microelectrode. Time dependent DO change was also monitored in which the terminal ratio between PFC (H_2O) and H_2O was 1:1.

In vitro cytotoxicity. Cytotoxicity was assessed by AlarmBlue assay and calcein-AM / propidium iodide (PI) staining. Firstly, we evaluated the cytotoxicity of PDT under hypoxic and normoxic conditions. Briefly, the CT26 cells (5 x 10^3 cells per well) were treated with PNPs with different concentrations for 2 h under different hypoxia atmosphere (1%, 21% kpa O_2). Then cells were immediately exposed to laser (808 nm, 400 mW cm^{-2}) for (30 s x 2) with an interval of 1 minute. After co-incubation for 2 h, the drugs were removed and fresh culture medium was added. Finally, the fluorescence intensity of AlarmBlue assay was measured (Ex/Em = 530/585 nm). To verify the cytotoxicity of TPZ under hypoxia atmosphere, the protocol was similar as above. The cells were treated with free TPZ at different concentrations for 24 h under different hypoxic atmosphere (1%, 10%, 21% O_2). Then the CT26 cells were mixed with the solution which consists of alarma blue (10μL) and fresh medium (100 μL) each well, these cells were incubated for another 2 h at 37°C and 5% CO_2 atmosphere. Finally, the fluorescence intensity was measured using a fluorescence microplate. The enhanced cytotoxicity of TPZ chemotherapy by PNPs in vitro was also assessed with the similar treatments of TPZ. For calcein-AM and PI assesses, the same treatments: (4 μg mL^{-1} IR780, 3% v/v PFC) were employed to detect live/dead cells, cells were stained with calcein-AM and PI for 15 min. Fluorescence images of cells were captured from the fluorescence microscope (Nikon, Japan) at an excitation of 535 nm and an emission of 600-680 nm.

In vitro blood clot retraction. Blood clot retraction was employed to evaluate the influence of PFCs on platelet aggregation. Briefly, mice (*n*=7) was

intravenously injected with PFTBA@HSA and saline. Blood was taken from eyes at 10 h post injection and centrifuged at 800 xg min^{-1} for 10 min, and the upper platelet-rich plasma was taken. The remaining samples was then centrifuged at 3000 xg min^{-1} for 10 min, and the lower layer was employed as the erythrocyte layer. 200 μL platelet-rich plasma and 5 μL of red blood cells were diluted to 1 mL with Tyrodes-HEPES. Then those samples was added with 1U mL^{-1} (100 U mL^{-1} add 10 μL) of thrombin and inserted with a capillary. Serum and clotting weight was measured after they were placed at room temperature for 90 min.

In vivo fluorescence imaging. For fluorescence imaging, Balb/c bearing CT26 mice were intravenously injected with 200 μL PNPs (1.4 mg kg^{-1} IR780, 20% v/v PFC). Then, fluorescence images of mice were captured by CRI maestro system using a 710 nm excitation wavelength. The images and fluorescence intensity were calculated by the maestro software. All of the mice were euthanized after 36 h imaging with the lower fluorescence intensity of 36 h than that of 24 h.

Detection of ROS in vivo. Carboxy-H_2DCFDA was employed to measure the generation of ROS in vivo. It acts as a fluorescent probe (green) in the presence of ROS. Briefly, 200 μL samples (1.4 mg kg^{-1} IR780; 20% v/v PFTBA,) were intravenously injected into the CT26 tumour-bearing mice. At 24 h post injection, all of those mice were intratumorally injected with 50 μL Carboxy-H_2DCFDA (25 mM). Then those mice were irradiated with laser (808 nm, 400 mW cm^{-2}) for 5 min. The fluorescence emission spectrum of carboxy-H_2DCFDA (Ex/Em = 495/529 nm) and Hoechst 33342 (Ex/Em = 350/461 nm) were immediately performed on a fluorescence microscope (Nikon, Japan).

Photothermal effect of PNPs in vivo. To evaluate the photothermal effect of PNPs in vivo, the CT26 tumor-bearing mice were intravenously injected with samples (1.4 mg kg^{-1} for IR780,) when the tumour volumes reached 80~100 mm^3. At 24 h post injection, all the mice were exposed to 808 nm laser irradiation (400 mW cm^{-2}) for a total of 5 min. The IR images were captured by

IR imaging devices (FOTRIC) at 0, 1, 3 and 5 min.

Bio-distribution of TPZ in CT26 tumour.

TPZ@PLGA was synthesized according to previous study. When tumour volume was reach around 150-200 mm^3, PNPs were first intravenously injected into mice at the dose of 200 μL (1.4 mg kg^{-1} IR780; 20% PFC, v/v). TPZ@PLGA was then injected into mice intravenously 5 h post PNPS injection. Mice were sacrificed 10 h after TPZ@PLGA injection. 0.1 g tumour tissues were homogenized. 1 mL normal saline was added to the wet tissue and homogenized for 5 min. The samples (400 μL) were transferred to a 1.5 mL eppendorf tube and mixed with 800 μL methanol. The mixture was vortexed well and then centrifuged for 10 min at 15,000 xg. The supernatant was injected to HPLC for analysis.

Assessment of tumour vessels post PDT.

The anti-mouse CD31 antibody (Bio-legend, 102508) was used to measure the densities of tumour vessels past PDT. Briefly, 200 μL samples (PNPs [1.4 mg kg^{-1} IR780; 20% v/v PFTBA], ONPs, NPs or saline) was injected intravenously into the mice when the CT26 tumour diameter reached 6-8 mm. Then, the tumours were irradiated (808 nm, 400 mW cm^{-2}) for 5 min at 24 h post-administration. 3 h later, the mice were sacrificed, and the tumour slices was prepared for ex vivo immunofluorescence staining of tumour vessels (anti-mouse CD31 antibody, dilution at 1:500). Finally, the sections were imaged using a digital microscope (Nikon, Japan).

PNPs enhanced TPZ chemotherapy in vivo. Male balb/c mice bearing 60-80 mm^3 CT26 tumours were randomly divided to 4 groups (*n* = 6-7). The treatment scheme was as listed: 1. saline, 2. PNPs, 3.TPZ, 4. PNPs plus TPZ. PNPs (1.4 mg kg^{-1} IR780; 20% v/v PFTBA) were injected intravenously and TPZ (1mg kg^{-1}) was injected intratumourally 3 h before laser exposure. All tumours were irradiated by laser (808 nm, 400 mW cm^{-2}) for 5 min at 24 h post administration of PNPs. The lengths (L) and widths (W) of tumours were recorded every two days using a digital caliper, volume (V) was calculated according to this formula: $V = L \times W^2/2$. Relative tumour volume was calculated as V/V_0 (V_0 is the initial tumour volume before treatments). Mice were sacrificed at day 12 and tumours from each group were weighted. Liver, spleen, kidney, heart, and lung from each group were collected, fixed with 4% paraformalclehyde solution for a day, mounted with paraffine, sliced, stained with hematoxylin and eosin (H&E), acquired by a digital microscope (Nikon, Japan). The therapeutic effects of those different treatments were also evaluated by H&E and TUNEL assays, but mice from each group were sacrificed 2 days after laser exposure.

References

1. Lin, H. et al. Feasibility study on quantitative measurements of singlet oxygen generation using singlet oxygen sensor green. *J Fluoresc* **23**, 41-47 (2013).
