# Supplementary material for: Perfluorocarbon regulates the intratumoural environment to enhance hypoxia-based agent efficacy
Source: Nat Commun. 2019 Apr 5;10:1580. doi: 10.1038/s41467-019-09389-2 (PMC6450981; doi:10.1038/s41467-019-09389-2)
Supplement: Supplementary file 3 — Reporting Summary [file 41467_2019_9389_MOESM3_ESM.pdf]

nature research

Corresponding author(s): Jinhui Wu

Reporting Summary

Nature Research wishes to improve the reproducibility of the work that we publish. This form provides structure for consistency and transparency in reporting. For further information on Nature Research policies, see Authors & Referees and the Editorial Policy Checklist.

Statistical parameters

When statistical analyses are reported, confirm that the following items are present in the relevant location (e.g. figure legend, table legend, main text, or Methods section).

n/a	Confirmed	
☐	☒	The exact sample size (*n*) for each experimental group/condition, given as a discrete number and unit of measurement
☐	☒	An indication of whether measurements were taken from distinct samples or whether the same sample was measured repeatedly
☐	☒	The statistical test(s) used AND whether they are one- or two-sided<br>*Only common tests should be described solely by name; describe more complex techniques in the Methods section.*
☒	☐	A description of all covariates tested
☒	☐	A description of any assumptions or corrections, such as tests of normality and adjustment for multiple comparisons
☐	☒	A full description of the statistics including central tendency (e.g. means) or other basic estimates (e.g. regression coefficient) AND variation (e.g. standard deviation) or associated estimates of uncertainty (e.g. confidence intervals)
☒	☐	For null hypothesis testing, the test statistic (e.g. *F*, *t*, *r*) with confidence intervals, effect sizes, degrees of freedom and *P* value noted<br>*Give P values as exact values whenever suitable.*
☒	☐	For Bayesian analysis, information on the choice of priors and Markov chain Monte Carlo settings
☒	☐	For hierarchical and complex designs, identification of the appropriate level for tests and full reporting of outcomes
☒	☐	Estimates of effect sizes (e.g. Cohen's *d*, Pearson's *r*), indicating how they were calculated
☐	☒	Clearly defined error bars<br>*State explicitly what error bars represent (e.g. SD, SE, CI)*

Our web collection on statistics for biologists may be useful.

Software and code

Policy information about availability of computer code

Data collection	Excel
Data analysis	Origin 8/GraphPad Prism 5

For manuscripts utilizing custom algorithms or software that are central to the research but not yet described in published literature, software must be made available to editors/reviewers upon request. We strongly encourage code deposition in a community repository (e.g. GitHub). See the Nature Research guidelines for submitting code & software for further information.

Data

Policy information about availability of data

All manuscripts must include a data availability statement. This statement should provide the following information, where applicable:

- Accession codes, unique identifiers, or web links for publicly available datasets
- A list of figures that have associated raw data
- A description of any restrictions on data availability

The authors declare that the main data supporting the findings of this study are available within the article and its Supplementary Information. Extra data are available from the corresponding author upon request.

Field-specific reporting

Please select the best fit for your research. If you are not sure, read the appropriate sections before making your selection.

☒ Life sciences ☐ Behavioural & social sciences ☐ Ecological, evolutionary & environmental sciences

For a reference copy of the document with all sections, see nature.com/authors/policies/ReportingSummary-flat.pdf

Life sciences study design

All studies must disclose on these points even when the disclosure is negative.

Sample size	The sample size was determined by allowable error size and accuracy, and resources.
Data exclusions	No data were excluded from the analyses.
Replication	Confirm the repeatability of the experimental findings.
Randomization	The samples were randomly grouped.
Blinding	The investigators were blinded to group allocation during data collection and analysis.

Reporting for specific materials, systems and methods

Materials & experimental systems

n/a	Involved in the study
☒	☐ Unique biological materials
☐	☒ Antibodies
☐	☒ Eukaryotic cell lines
☒	☐ Palaeontology
☐	☒ Animals and other organisms
☒	☐ Human research participants

Methods

n/a	Involved in the study
☒	☐ ChIP-seq
☒	☐ Flow cytometry
☒	☐ MRI-based neuroimaging

Antibodies

Antibodies used

The following primary antibodies were used for western blotting. They are listed as antigen first, followed by supplier, catalog number and clone/lot number as applicable.
1) Anti HIF1-α antibody, Abcam, cat. no. ab51608;
2) Anti β-actin antibody, Seville, cat. no. GB13001-1;
3) Goat anti-rabbit antibody, cat. no. GB23301;
The following primary antibodies were used for immunofluorescence. They are listed as antigen first, followed by supplier, catalog number and clone/lot number as applicable.
1) Anti HIF1-α antibody, Thermal Fisher, cat. no. MA5-16009;
2) Anti Glut-1 antibody, Proteintech, cat. no. 21829-1-AP;
3) Anti CD31 antibody, Biolegend, cat. no. 102508;
4) Anti-Salmonella antibody, Abcam, cat. no. ab69253.

Validation

All antibodies were verified by the supplier and each lot has been quality tested.

Eukaryotic cell lines

Policy information about cell lines

Cell line source(s)	The mouse CT26 colorectal cancer cells, 4T1 breast cancer cells
Authentication	All the cells were purchased from China Type Culture Collection (CTCC) obtained from the American Type Culture Collection (ATCC).

Mycoplasma contamination	Cells were routinely tested for mycoplasma contamination using MycoSET Mycoplasma real-time PCR detection Kit.
Commonly misidentified lines (See ICLAC register)	No misidentified lines

Animals and other organisms

Policy information about studies involving animals; ARRIVE guidelines recommended for reporting animal research

Laboratory animals	male BALB/c mice, 5 weeks old.
Wild animals	The study did not involve wild animals.
Field-collected samples	The study did not involve any samples collected from field.
