# Supplementary material for: Perfluorocarbon regulates the intratumoural environment to enhance hypoxia-based agent efficacy
Source: Nat Commun. 2019 Apr 5;10:1580. doi: 10.1038/s41467-019-09389-2 (PMC6450981; doi:10.1038/s41467-019-09389-2)
Supplement: Supplementary file 2 — Peer Review File [file 41467_2019_9389_MOESM2_ESM.docx]

**Point-by-point response**

**Point-by-point response 1^st^:**

**REVIEWER #1**

*In this manuscript, the authors prepared a perfluorocarbon based nano photosensitizer (nano-PS), which was confirmed to be an efficient oxygen nano-sponge by utilizing its efficient oxygen absorption ability. By synergistically utilizing such efficient oxygen absorption ability, the authors demonstrated that the tumors post photodynamic treatment with such nano-PS showed a long-term elevated hypoxia status, which would favor the therapeutic efficacy of those hypoxia based agents such as anaerobic bacteria and bioreductive prodrugs. The similar treatment strategy by combining oxygen exhausting photodynamic treatment with bio-reductive prodrugs has been widely reported in the past several years as shown in references^1, 2, 3, 4^. Moreover, there are a number of major concerns as listed below. The clinical value of this technique has not been well demonstrated.*

**Answer:**

We are afraid that the reviewer #1 may not understand the [innovation](javascript:;) of our work. These references^1, 2, 3, 4^ cited by reviewer#1 are all based on the photodynamic effect of photosensitizers. These strategies must rely on the excitation and maintenance of laser irradiation.

Our manuscript reported the use of perfluorocarbon materials to simultaneously improve tumour permeability and hypoxia, which is not dependent on photosensitizers and irradiation, and has never been reported before.

1. *The authors should confirm whether such* *perfluorocarbon based nano-PS itself could synergistically enhance the treatment effect of TPZ and VNP20009 bacteria or not. A lot more in vivo control groups are needed in Figure 4 and Figure 5. The current in vivo data can only demonstrate that PDT is able to enhance the efficacy of HBA – that is already well known.*

**Answer:**

①In Fig. 2, data demonstrated that PNP can improve tumor permeability and enhance tumor hypoxia alone; In Fig. 3, data further demonstrated that PNP can be enhanced by Oxy-PDT mechanisms reported previously by us. Therefore, it’s no need to add extra control groups in Fig. 4 and 5.

②Many thanks for your suggestion. The same observation period (stopped at day 9~11) was common in nowadays PDT reports on high impact journals (such as day 11 on *Nature Medicine*^5^, day 9 on *ACS NANO*^6^ and day 10 on *Advanced Materials*^7^).

2. *In Figure 4b, only small* *differences between ‘PNPs + laser’ and ‘TPZ + PNPs+ laser’ could be observed. This seems to be contradictory to the in vivo data.*

**Answer:**

This phenomenon is reasonable and partly due to these reasons: 1.The different times of laser irradiation - *in vivo* for 5 min and *in vitro* for 1 min (30 s * 2 times); 2. Different initial oxygen pressures in tumor (about 0-30 mmHg) and *in vitro* (about 160 mmHg); 3. Concentrations of prodrug-activating reductases, which could turn TPZ into toxic radicals, is much higher *in vivo* than those *in vitro*.

*3. In the Introduction section, the authors described that “**Abnormal vasculature and dense tissue in tumour severely limit the intratumoural drug penetration, thus those anaerobic drugs are always obstructed in aerobic areas nearby tumour vessels rather than reaching hypoxic cells^1, 2, 3, 4^”. However, these four reference papers are describing the combination therapy of bio-reductive prodrugs with* *those oxygen-exhausting treatments. Therefore, the authors' claim here is not evidenced by previous studies based on listed.*

**Answer:**

The contents are indeed mentioned in those references (for example, in their introduction and discussion), and now we have also added new reference:

1. Castro, Camila Irene, and Juan Carlos Briceno. "Perfluorocarbon‐based oxygen carriers: review of products and trials." Artificial organs 34.8 (2010): 622-634.

2. von der Hardt, Katharina, et al. "Comparison of aerosol therapy with different perfluorocarbons in surfactant-depleted animals." Critical care medicine 32.5 (2004): 1200-1206.

3. Young, Lawrence H., et al. "Metabolic and functional effects of perfluorocarbon distal perfusion during coronary angioplasty." American Journal of Cardiology 65.15 (1990): 986-990.

4. Song, Xuejiao, et al. "Ultrasound triggered tumor oxygenation with oxygen-shuttle nanoperfluorocarbon to overcome hypoxia-associated resistance in cancer therapies." Nano letters 16.10 (2016): 6145-6153.

Thanks for your reminding.

*4. In line 18(page 11), the authors described that “Characterizations including electron microscopy image, dynamic light scattering and UV-vis spectra are all available (Supplementary Fig. 1, 2)”. I suggest the authors to describe the results in details.*

**Answer:**

Thanks for your suggestion; we have described those characterizations including electron microscopy image, dynamic light scattering and UV-vis spectra (Supplementary Fig. 1, 2) more detailed in the revision.

*5. In Figure 3 and 4, I suggest the authors to provide the oxygen-absorbing ability of oil as a reference.*

**Answer:**

Reviewer# 1 may not notice that lower oxygen-absorbing ability of ONPs was monitored in Fig. 1b and 1c compared with PNPs.

*6. In line 166-167, the authors described that “We also observed that ^1^O_2_ production by PNPs at 1% O_2_ partial pressure was almost equal to those at 21% O_2_ partial pressure by control groups, suggested an unique synergistic PDT effect of PFTBA in PNPs.” However, according the result shown in Figure 2a&b, it was shown that the SOSG fluorescence intensity of PNPs* *(2 μg/mL) upon exposed to laser under normaxia was about 10000, while that was less than 3000 upon irradiated under hypoxia. Therefore, I think that these two results are greatly different instead of ‘almost equal’.*

**Answer:**

We fell sorry that reviewer#1 may misread the sentence ‘those at 21% O_2_ partial pressure by control groups’. This ambiguous sentence has now been modified in the revision.

*7. I want to see if the tumors of mice with injection of such IR780 containing nano-PS would show obvious temperature increase upon being subjected to 808 nm laser irradiation at 400 mW/cm^-2^ for 5 min.*

**Answer:**

IR thermal images of CT26-tumour-bearing mice injected with PNPs or NPs under the 808 nm laser (0.4 W/cm^2^ for 5 min) irradiation was used to reflect tumour temperature. As it shown, temperature rise will not cause photothermal therapy theoretically, because temperature was controlled below 42℃^8, 9^.

**Fig. 1** IR thermal images of CT26-tumour-bearing mice injected with PNPs and NPs under the 808 nm laser (0.4 W/cm^2^ for 5 min) irradiation.

*8. For in vivo treatment, the TPZ was* *intratumorally injected-this has very limited clinical value. How about the combination treatment effect if TPZ was intravenously injected?*

**Answer:**

Intratumoral injection does not limit the use of TPZ. In addition, in many clinical trials, drug was intratumorally injected into patients instead of intravenous injection (For example, *Cell* 170.6 (2017): 1109-1119.). We also demonstrated intravenous administration experiments for VNP20009.

*9. In line 392-393, the authors described that “To remove free IR780, NPs were hyper-filtrated by a 30 kDa millipore filter.” IR780 molecules are hydrophobic and may form large aggregated in the aqueous solution. Therefore, it might be difficult to remove IR780 by using such 30 kDa millipore filter. Please double check*.

**Answer:**

Many thanks for your suggestion. We had made a check about the result. The aggregated ir780 were firstly removed by filter and it was also gotten out by ultrafiltration. Comparing with the result from molecular sieve, there is no difference. Therefore, the free IR780 could be removed by the 30 kDa millipore filter.

*10. The experimental process of Figure S9 should be described in details.*

**Answer:**

Thanks for your suggestion. More detailed experimental process of Figure S9 was added in the supplement file.

**Reviewer #2:**

*This study speaks to a growing are of study on the potential of nanoparticles based model therapeutic agents to modify local tumour environments and enhance the effectiveness of photodynamic therapy when it is restrained by the hypoxic tumour environment. The authors approach the reasonable hypothesis that hypoxia based agent (HBA) action is also restricted by both the dynamics of hypoxia generation and the ability of a candidate agent to penetrate the low vascular tumour microenvironment. Their approach is reasoned and logical, demonstrating sequence in the levels of study that are required to make their conclusions. In some ways the technical details (which appears well justified and reported) are less important than the rationalization of the approach and this readers would find interesting. There are multiple and recent publications that touch upon different aspects of the principles drawn upon in their study. The authors have been selective in their referencing to wider studies but within the format this is reasonable. They employ a range of assays that are accepted in practice. However each will have its drawback and can be criticised as such but still represent typical approachs for the analysis of tissue damage, hypoxic response, cell death, local hypoxic level, etc.*

*The key points of interest for readers are: the convincing demonstration of the O_2_ absorbing during and after photodynamic treatment; the slightly surprising observation that even that PNPs alone can increase the drug permeation without laser triggering; PNPs mediated PDT can effectively increase the cytotoxicity of TPZ. These observations alone give confidence in their wider conclusions.*

**Answer:**

Many thanks for your interesting and approval for our manuscript. Thanks again for your kind recommendation!

*The potential for clinical application is another matter. Informed readers will be aware of all of the caveats - here the future applications are less certain. A balanced view of the current situation for HAPs should be refereed to (Br J Cancer. 2016 May 10;114(10):1071-7. doi: 10.1038/bjc.2016.79. Epub 2016 Apr 12. Hypoxia-activated prodrugs: paths forward in the era of personalized medicine. Hunter et al.).* *On the face of it the advantageous potential for enhancing tumour hypoxia as a strategy at its fundamental level appears naïve, given all of the clinical and pre-clinical studies that indicate enhanced hypoxia promotes tumour aggression and immune suppression within the microenvironment that further promote tumour spread. Thus manoeuvres enhance hypoxia are potentially dangerous and the modification of local permeability will have unknown effects on tumour spread. The introduction of the PDT dimension is another matter and here this submission should pique the interest of reader - here the critical feature is the tumour indication and the balance between effective destruction of cells within a hypoxic environment and the pro-progression effects of sustained hypoxia. In general the paper is promoting the approach described (valid within the constraints of the study) but tend to ignore the negative aspects and noted above. This the authors should correct and provided the readership with that backdrop. The authors note the potential for reduction in doses of a hypoxia-dependent to minimise of target effects and this is a clinically attractive aspect.*

**Answer:**

Many thanks for your kind recommendation. In the revision, we have discussed the issues about the balanced view of enhancing hypoxia for tumor therapies or tumour aggression. Enhanced hypoxia may promotes tumour aggression and immune suppression within the microenvironment that further promote tumour spread^10^. But the pro-progression effects of pre-hypoxia treatment could be reversed by those combinations of pre-hypoixa therapy and HBA^4, 11^. This combination may suppress hypoxia-stimulated tumour aggression and spreading and much down-regulated expressions of Snail transcription factor responsible for cancer invasion and metastasis^12^. We believe the revision can arouse a broad interest of Nature communications’ readers.

**Point-by-point response 2^nd^:**

*Reviewer #1 (Remarks to the Author):*

*In this revised manuscript, the authors have reorganized the manuscript in a reasonable way. In this revised manuscript, the authors clearly demonstrated that the as-prepared perfluorocarbon nanoparticle combined with laser activated photodynamic therapy together could create a long lasting hypoxic microevironment for the activation of those hypoxia based agents. Overall, this manuscript could be considered for acceptance after further revisions.*

**Answer:**

Thanks very much for your recognition of our work.

1. *I suggest the authors to provide the results of in vivo blood test and blood biochemistry assay data for mice with salmonella VNP20009 injection.*

**Answer:**

In the revised manuscript, we have evaluated the blood biochemistry and hematologic indexes after intravenous administration of VNP20009. As shown in **Fig. 5f**, the blood biochemistry of those VNP20009 treated mice varied significantly on day 1, especially the AST and ALT, ~ 2.94 and ~2.54 times compared with saline. But no long-term side effects of VNP20009 could be found on day 7 post injection. These results indicate that intravenous injection of VNP20009 at a dose of 5∙10^6^ CFU per mouse is feasible.

Modifications in manuscript were as bellows: Line 343-344, Page 16; Line 345-351, Page 17; Fig.5 f; Line 869-872, Page 36.

2. *I am curious about why such perfluorocarbon nanoparticles would not release the adsorbed oxygen under the reduced oxygen gradients. Please provide additional data to further verify and explain this phenomenon.*

**Answer:**

As reviewer #1 said, intravenously injected PNPs may optimize tumour hypoxia by releasing the adsorbed oxygen under the reduced oxygen gradients. But in our study, since PFTBA is combined with PDT, the oxygen in PFTBA and tumour is consumed, making them more hypoxic. The anoxic PFTBA then works as an oxygen absorber and prevent the recovery of tumour oxygen tension for over 24 h, as shown in **Fig. 3f-i**. Besides, intratumour injected PNPs which were previously deoxygenated thus would not deliver extra oxygen into tumour, as shown in **Fig. 2e, f**.

Thanks again for recommending our article.

*Reviewer #3 (Remarks to the Author):*

*This manuscript* appears to represent sound science *but the poor grammatical style* *renders the document, in many instances, unintelligible and offers limited capacity to ascertain the true meaning the authors intend to convey. The authors are encouraged to seek some editorial assistance prior to submission. It is NOT the task of the peer reviewer to wade through the English to interpret and correct. There are professional individuals and software able to provide these services and the authors are advised to seek assistance in order to present their significant efforts in the best light possible. The grammatical challenges are sufficiently great that I am only prepared to review the document in full once they are addressed. I would encourage the authors to correct the language and resubmit. By way of example, I illustrate some of the grammatical and technical issues of note in the abstract and introduction.*

**Answer:**

We're sorry for our bad writing. The manuscript has been modified according to reviewer’s comments. The abstract and introduction section have been re-written. The revised manuscript was further improved and revised with the help of *Springer Nature Author Services*. Thanks for your kind suggestions.

*23-28; Definition of the therapeutic challenge is poorly articulated. For example, what is a“traditional tumour regulating method”? Recommend rewriting.*

**Answer:**

We have rewritten the corresponding content. Modifications in manuscript were as bellows: Line 24-28, Page 2.

*29; What is meant by permeable?*

**Answer:**

We have rewritten the corresponding content. What we want to express is that PNPs can make drug easier to accumulate in tumor. Modifications in manuscript were as bellows: Line 29-32, Page 2.

*31; define ‘strengthened’ in relation to hypoxia. Avoid ambiguous use of adjectives.*

**Answer:**

We have rewritten the manuscript according to your comments. Modifications in manuscript were as bellows: Line 29-32, Page 2.

*35; ‘…great potential to relight the effect of hypoxia based drugs’ is colloquial language. The meaning is unclear and more formal language is recommended.*

**Answer:**

We have rewritten the manuscript according to your comments. Modifications in manuscript were as bellows: Line 32-34, Page 2.

*38 ‘bring a new island’ is colloquial language. The meaning is unclear.*

**Answer:**

We have rewritten the corresponding content. Modifications in manuscript were as bellows: Line 37-38, Page 3.

*39; ‘an oxygen negatively-related cytotoxic effect’. The meaning is unclear/ambiguous.*

**Answer:**

We have rewritten the manuscript according to your comments. Modifications in manuscript were as bellows: Line 37-38, Page 3.

*44; ‘failed due to the unsatisfied efficacy’ is not grammatical. (…failed due to
unsatisfactory efficacy)*

**Answer:**

We have rewritten the manuscript according to your comments. Modifications in manuscript were as bellows: Line 43-44, Page 3.

*45; ‘bad penetration to hypoxic tumour’ is not grammatical. (…restricted penetration to hypoxic tumour regions).*

**Answer:**

We have rewritten the manuscript according to your comments. Modifications in manuscript were as bellows: Line 43-44, Page 3.

46-7; ‘anaerobic drugs are always obstructed in aerobic areas nearby tumour vessels’. The meaning is unclear. Why? How?

**Answer:**

We have rewritten the corresponding content. What we want to express is that tumour periphery is usually less permeable than the hypoxic core and limit further drug penetration^13^.Modifications in manuscript were as bellows: Line 44-47, Page 3.

49-50; ‘enhancing tumour permeability is an effective approach to activate HBA’. Possibly true in some circumstance, but recommend use of less emphatic language.

**Answer:**

We have rewritten the corresponding content. Modifications in manuscript were as bellows: Line 47-48, Page 3.

53; ‘However, it’s embarrassed that the increased drug penetration is accompanied with the re-perfusion of O_2_’. It is not possible for a tumour to become embarrassed.

**Answer:**

We have rewritten the corresponding content. Modifications in manuscript were as bellows: Line 51-54, Page 3.

57; ‘but these laser-dependent regulations can't be maintained after laser was removed.’ Define meaning of ‘regulations’ (consider alternative word). The contraction “can’t” should be avoided in formal (scientific) language (use cannot). Generally poor grammatical structure.

**Answer:**

We have rewritten the corresponding content. Modifications in manuscript were as bellows: Line 56-58, Page 3; Line 59, Page 4.

59; ‘synergistically relight HBA's effect’. Meaning?

**Answer:**

We have rewritten the corresponding content. What we want to express is that PNP can simultaneously enhance permeability and hypoxia within tumour to make HBA more effective. Modifications in manuscript were as bellows: Line 59-61, Page 4.

*60; ‘To regulate tumour environment towards the drug permeable & hypoxia, two opposite directions, herein we reported that….’ The abstract and introduction appear predicated on the hypothesis that efficient drug diffusion and greater hypoxia are in opposition. However, this argument is not well made and should be improved upon.*

**Answer**:

Many thanks for the helpful comments. We apologize for unclearly describing the argument between drug diffusion and oxygen diffusion. The corresponding content has been rewritten. Modifications in manuscript were as bellows: Line 62-65, Page 4.

78; delete ‘kind of’. Unnecessary ambiguity.

**Answer:**

We have rewritten the manuscript according to your comments. Modifications in manuscript were as bellows: Line 81-83, Page 5.

*83; the average particle diameter of PNPs were determined as 160 nm. This should be raised in discussion section as particle diameter is a critical aspect of extravascular penetration. E.g. see Nature Communications volume 9, Article number: 1410 (2018) and references therein.*

**Answer:**

Thanks for the helpful suggestion. We have cited this reference and discussed the potential influences of particle size. Modifications in manuscript were as bellows: Line 400-405, Page 19.

93; tumor. English spelling required for consistency.

**Answer:**

We have rewritten the manuscript according to your comments. We have replaced all “tumor” by “tumour”.

93; High oxygen binding capacity of PFTBA may fit this goal.

**Answer:**

The corresponding content has been rewritten. Modifications in manuscript were as bellows: Line 98-99, Page 5.

*102; what is the mechanism of oxygen consumption by PNP? Explain.*

**Answer:**

The oxygen consumption was made by PNP-based PDT and the oxygen absorption of PNP. First, the oxygen consumption was achieved by PNP-based photodynamic therapy (PDT). Synergism between the co-encapsulated photosensitizer and PFTBA enable PNPs to exert a superior photodynamic effect compared to ordinary photosensitizers. Based on the unique affinities between O_2_/^1^O_2_ and PFTBA, with irradiation, the PNPs can efficiently convert tumour O_2_ into cytotoxic ^1^O_2,_ resulting in oxygen consumption. Second, PFTBA has a much higher oxygen solubility than water. Compared with the tumour stroma, it can absorb more oxygen to inhibit the recovery of tumour oxygen partial pressure after PDT depleted the intratumoural oxygen. Modifications in manuscript were as bellows: Line 370-376, Page 18.

*Of note, the authors claim PNPs enhances drug permeability in tumour issue (ln 115). However it is noted that tirapazamine (TPZ) is administered by direct intratumoural injection (ln 470) into very small tumours of 60-80 mm^3^ (ln 466). Whilst efficacy is observed it is difficult to concur with the conclusion that delivery of TPZ in the tumour microenvironment is optimised by PNP pretreatment since intravenous drug administration is avoided.*

**Answer:**

Many thanks for the helpful comments. In the revised manuscript, we evaluated both the TPZ penetration and efficacy of therapy by PNPs pretreatment through **intravenous administration**. Also we replace the previous intratumoural data and figures by intravenous data in this revised manuscript. We sincerely hope our modified paper and experimental supplements will meet with acceptance.

Modifications in manuscript were as bellows: Line 146-148, Page 7; Supplementary Fig. 9; Line 270-278, Page 13; Line 279-300, Page 14; Fig.4 c-f, Line 845-854, Page 35.

**Fig. 1** The distribution of TPZ in tumour of CT26 bearing mice. TPZ was a**dministrated intravenously** at 5 h after PNPs were i.v. injected into mice. At 10 h post intravenous administration of TPZ, mice were sacrificed and the level of TPZ in different tissues was evaluated. Values are the mean ± s.d. (n = 4 or 5).

**Fig. 2** (a) Tumour growth curves of treated mice. Both PNPs and TPZ were injected intravenously into mice at day 1, 3 and 5. Tumours in the group of 3, 4, 5 and 6 were irradiated three times by laser (808 nm, 400 mW∙cm^-2^) for 5 min at 24 h post administration of PNPs. (b) Normalized averages of tumour weights at day 13. (c) Changes of body weights. (d) H&E and TUNEL stained CT26 slices. Samples were collected from different groups at day 8 post administration (scale bars, 100 µm). Values are the mean ± s.d. (n ≥6 mice per group). ******p* < 0.05, *******p* < 0.01, ********p* < 0.001.

**Fig. 3** Photographs of mice from different groups after corresponding treatments, including control (saline), free TPZ, ONPs, PNPs, TPZ plus ONPs and TPZ plus PNPs. TPZ was **intravenously injected into mice** 3 h before laser irradiation. All of the groups were exposed to laser (808 nm, 400 mW∙cm^-2^) for 5 min at 24 h after intravenous administration of PNPs.

**References**

1.	Feng L*, et al.* Theranostic Liposomes with Hypoxia-Activated Prodrug to Effectively Destruct Hypoxic Tumors Post-Photodynamic Therapy. *ACS nano* **11**, 927-937 (2017).

2.	Liu Y*, et al.* Hypoxia Induced by Upconversion-Based Photodynamic Therapy: Towards Highly Effective Synergistic Bioreductive Therapy in Tumors. *Angew Chem Int Ed Engl* **54**, 8105-8109 (2015).

3.	Qian C*, et al.* Anaerobe-Inspired Anticancer Nanovesicles. *Angew Chem Int Ed Engl* **56**, 2588-2593 (2017).

4.	Wang Y*, et al.* Tumor-Penetrating Nanoparticles for Enhanced Anticancer Activity of Combined Photodynamic and Hypoxia-Activated Therapy. *ACS Nano* **11**, 2227-2238 (2017).

5.	Idris NM, Gnanasammandhan MK, Zhang J, Ho PC, Mahendran R, Zhang Y. In vivo photodynamic therapy using upconversion nanoparticles as remote-controlled nanotransducers. *Nature medicine* **18**, 1580-U1190 (2012).

6.	Jin CS, Lovell JF, Chen J, Zheng G. Ablation of Hypoxic Tumors with Dose-Equivalent Photothermal, but Not Photodynamic, Therapy Using a Nanostructured Porphyrin Assembly. *Acs Nano* **7**, 2541-2550 (2013).

7.	Liu YL, Ai KL, Liu JH, Deng M, He YY, Lu LH. Dopamine-Melanin Colloidal Nanospheres: An Efficient Near-Infrared Photothermal Therapeutic Agent for In Vivo Cancer Therapy. *Adv Mater* **25**, 1353-1359 (2013).

8.	Cheng L, Wang C, Feng L, Yang K, Liu Z. Functional nanomaterials for phototherapies of cancer. *Chem Rev* **114**, 10869-10939 (2014).

9.	Sherlock SP, Tabakman SM, Xie L, Dai H. Photothermally enhanced drug delivery by ultrasmall multifunctional FeCo/graphitic shell nanocrystals. *ACS Nano* **5**, 1505-1512 (2011).

10.	Quail DF, Joyce JA. Microenvironmental regulation of tumor progression and metastasis. *Nat Med* **19**, 1423-1437 (2013).

11.	Liu Y*, et al.* Radiation-/hypoxia-induced solid tumor metastasis and regrowth inhibited by hypoxia-specific upconversion nanoradiosensitizer. *Biomaterials* **49**, 1-8 (2015).

12.	Jin H*, et al.* Snail is critical for tumor growth and metastasis of ovarian carcinoma. *Int J Cancer* **126**, 2102-2111 (2010).

13.	Brown JM, Wilson WR. Exploiting tumour hypoxia in cancer treatment. *Nat Rev Cancer* **4**, 437-447 (2004).

**Point-by-point response 3^rd^:**

*Reviewer #1 (Expertise: Nanoparticles, TME, hypoxia therapy, Remarks to the Author):*

*In this revised manuscript, the authors have reorganized the manuscript in a reasonable way. In this revised manuscript, the authors clearly demonstrated that the as-prepared perfluorocarbon nanoparticle combined with laser activated photodynamic therapy together could create a long lasting hypoxic microenvironment for the activation of those hypoxia based agents. Overall, this manuscript could be considered for acceptance after further revisions.*

**Answer:**

Thanks very much for your recommending of our work.

1. *In this manuscript, I think that the question 2 “I am curious about why such perfluorocarbon nanoparticles would not release the adsorbed oxygen under the reduced oxygen gradients. Please provide additional data to further verify and explain this phenomenon” was not fully answered by the current reply. In my opinion, the PNPs, NPs and ONPs would cause different degrees of damage to tumour blood vessels, which are responsible for tumour oxygen supply, upon laser irradiation since these three types of NPs have different photosensitization capacity as indicated in the supplementary Figure 6. Therefore, it is hard to conclude that the more severe tumour hypoxia at 24 h post the treatment of PNPs + laser irradiation Fig. 3f-i is attributing to the strong oxygen absorbing capacity, while no the blood vessel damage induced blockade of tumour oxygen supply. So, the tumour blood vessel structure and perfusion profiles post various treatments should be evaluated.*

**Answer:**

Thanks for your kind concerns. We highly appreciate your in-depth discussion about the long-term hypoxia. We totally agree with you that it is caused by both photodynamic therapy (PDT) effect and the oxygen absorbing effect of PFTBA. Here are 3 points of our supplementary explanations.

1. As you mentioned, PNPs, NPs and ONPs have different PDT efficacy, in which PFTBA enables PNPs the strongest PDT efficacy to destroy tumour vessels and consume oxygen. We additionally evaluated the tumour blood vessel structure as suggested. As shown in **Response Fig. 1 (sFig. 16 in supplementary text)**, the blood vessels after PNP treatment are most severely damaged, which would further cause sustained hypoxia. In addition, as shown in **Response Fig. 2 (supplementary Figure 6)**, the ability of PNP to photodynamically consume oxygen is also the strongest. In summary, PFTBA enable PNP to cause more severe tumour hypoxia through enhancing PDT effect.
2. In addition to the above-mentioned PDT enhancement, PFTBA also strengthen tumour hypoxia through non-PDT approaches, especially by naturally absorbing tumour oxygen. As shown in **Response Fig. 3**, deoxygenated PNPs can decrease dissolved oxygen more than deoxygenated ONPs *in vitro*. As shown in **Response Fig. 4 （Fig. 2d-f in main text）**, deoxygenated PNP can directly result in more severe tumour hypoxia for over 24 h without PDT treatment *in vivo*. These data indicate that PFTBA can reduce the oxygen concentration of the external tumour tissue by oxygen absorption.
3. As shown in **Response Fig. 5 (Fig. 3e in main text)**, after the end of laser treated deoxygenation (0-5min), PNP still decrease and maintain the low intratumoural oxygen partial pressure, while those in ONP treated group recovered immediately (5-60min). We need to point out that, although it’s hard to distinguish how much PFTBA has contributed to the tumour hypoxia in **Fig. 3f-i** by enhancing PDT and absorbing oxygen after PDT respectively, the experiments above have proved that both approaches are effective.

I hope the above data and discussion have solved your doubts. Thanks again for your kind concerns. We have rewritten the responding content in revised manuscript. Modifications in manuscript were as bellows: Line 219-221, Page 11; Supplementary Fig. 16, line 130-135, Page s17; Line 348-357, Page s33.

Thanks again for recommending our article.

**Response Fig. 1** *Ex vivo* immunofluorescence images of tumour vessels post PDT. (a) The tumour vessels and nuclei were stained with anti-CD31 antibody (red) and DAPI (blue), respectively. (b) Quantification of blood vessel densities after irradiation. Data are presented as the mean ± s.e.m. (Saline, PNPs, NPs, n=5; ONPs, n=6).

**Response Fig. 2** Phosphorescence (PS) lifetime profile of phosphorescent molecular probe after PDT. The lower PO_2_, the longer the PS lifetime. The fact that PNPs produced longer PS lifetime indicated that a more hypoxic environment could be achieved by PNPs. Data are presented as the mean ± s.e.m.

**Response Fig. 3** *In vitro* changes of dissolved oxygen (DO) by deoxygenated PNPs and ONPs without PDT. Data are presented as the mean ± s.e.m. (n=3).

**Response Fig. 4** *In vivo* enhanced hypoxia by deoxygenated PNPs **without** PDT. (a) Changes of dissolved oxygen (DO) after the intratumoural injection of deoxygenated PNPs and ONPs. (b) Representative immunofluorescence images of HIF-1α staining at 24 h after the intratumoural injection of deoxygenated PNPs and ONPs (scale bar, 100 µm). (c) Quantification of HIF-1α expression (n=4). Data are presented as the mean ± s.e.m.

**Response Fig. 5** Changes in DO after PDT (808 nm, 400 mW∙cm^-2^) for 5 min. Data are presented as the mean ± s.e.m. (n=3).

*Reviewer #2 (Expertise: Hypoxia based agents, cancer therapy, Remarks to the Author):*

*I commend the authors for resubmitting a much improved manuscript which is suitable for publication. I believe the data presented represents new knowledge that will be of interest to readers of Nature Communications.*

Many thanks for your interesting and approval for our manuscript. Thanks again for your kind recommendation!

*I have a few minor comments;*

1. *63; indicate PFTBA is clinically approved, citing reference 25. This statement suggests PFTBA is FDA/EMA approved and marketed for human use; however this reference does not support the statement. Please clarify.*

**Answer:**

Many thanks for the helpful comments. The contents are indeed mentioned in the reference 25. The corresponding content is captured in the screenshot:

**Response Fig. 6** screenshot of corresponding content in reference. 25.

We have also added more references as followed:

1. Castro, Camila Irene, and Juan Carlos Briceno. "Perfluorocarbon based oxygen carriers: review of products and trials." Artificial organs 34.8 (2010): 622-634.

2. von der Hardt, Katharina, et al. "Comparison of aerosol therapy with different perfluorocarbons in surfactant-depleted animals." Critical care medicine 32.5 (2004): 1200-1206.

3. Young, Lawrence H., et al. "Metabolic and functional effects of perfluorocarbon distal perfusion during coronary angioplasty." American Journal of Cardiology 65.15 (1990): 986-990.

4. Song, Xuejiao, et al. "Ultrasound triggered tumouroxygenation with oxygen-shuttle nanoperfluorocarbon to overcome hypoxia-associated resistance in cancer therapies." Nano letters 16.10 (2016): 6145-6153.

Thanks for your reminding. Modifications in manuscript were as bellows: Line 58-62, Page 4.

2. *151; Define ‘intratumoural DO level’ on first use of term DO. Perhaps acronym stands for ‘dissolved oxygen’.*

**Answer**:

Many thanks for the helpful suggestion. We are sorry for unclearly describing the dissolved oxygen (DO) on first use of term DO. In the revised manuscript, we have defined the dissolved oxygen as DO.

Modifications in manuscript were as bellows: Line 147-148, Page 8.

3. *In discussion, it would be helpful for readers to summarise the potential challenges of laser irradiating non-superficial disease.*

**Answer**:

Many thanks for your kind recommendation. In the revision, we have discussed the issues about the potential challenges of laser irradiating non-superficial disease.

Modifications in manuscript were as bellows: Line 403-407, Page 19; Line 408-410, Page 20.

**Point-by-point response 4^th^:**

*REVIEWERS' COMMENTS:*

*Reviewer #1 (Expertise: Nanoparticles, TME, hypoxia therapy, Remarks to the Author): This work is now acceptable for publication.*

**Answer:**

Thanks very much for your recommending of our work.
